# Burden of respiratory syncytial virus in adults in the United Kingdom: A systematic literature review and gap analysis

**DOI:** 10.1111/irv.13188

**Published:** 2023-09-21

**Authors:** Tom Wilkinson, Steph Beaver, Malcolm Macartney, Eve McArthur, Vaishali Yadav, Annie Lied‐Lied

**Affiliations:** ^1^ Clinical and Experimental Sciences University of Southampton Faculty of Medicine Southampton UK; ^2^ National Institute for Health and Care Research Southampton Biomedical Research Centre Southampton UK; ^3^ Costello Medical London UK; ^4^ Janssen‐Cilag High Wycombe UK; ^5^ Costello Medical Cambridge UK

**Keywords:** adult, elderly, respiratory syncytial virus, respiratory tract infections, systematic review, United Kingdom

## Abstract

Despite the growing recognition of a potentially significant respiratory syncytial virus (RSV) disease burden in adults, relevant evidence in the United Kingdom (UK) is limited. This systematic literature review (SLR) aimed to identify the disease burden of RSV in UK adults, including certain high‐risk subgroups and existing evidence gaps. Published studies (2011 onwards) reporting epidemiological, economic and clinical burden outcomes in UK adults (≥15 years) with RSV were identified from indexed databases, including MEDLINE, Embase and the Cochrane library. High‐risk groups included elderly (≥65 years), immunocompromised, co‐morbid and co‐infected patients. Outcomes included RSV incidence/prevalence, mortality, clinical presentation and direct/indirect resource use/costs. Twenty‐eight publications on 28 unique studies were identified, mostly in general/respiratory indicator (*n* = 17), elderly (*n* = 10) and immunocompromised (*n* = 6) cohorts. Main outcomes reported in the general/respiratory indicator cohort were RSV infection incidence (seasonal/annual: 0.09–17.9%/6.6–15.1%), mortality (8,482 deaths/season) and direct resource use (including mean general practitioner [GP] episodes/season: 487,247). Seasonal/annual incidence was 14.6–26.5%/0.7–16% in high‐risk cohorts. Attributed to RSV in the elderly were 7,915 deaths/season and 175,070 mean GP episodes/season. Only two studies reported on co‐morbid cohorts. Clinical burden outcomes were only reported in general and immunocompromised patients, and no evidence was found in any cohort on indirect economic burden or RSV complications. Evidence captured suggests that RSV may have a substantial burden in UK adults. However, available data were limited and highly heterogenous, with further studies needed to characterise the burden of RSV in adults and to validate our findings.

## 1 INTRODUCTION

There are a number of respiratory viruses causing seasonal epidemics in the United Kingdom (UK). The most broadly studied and commonly known of these viruses is influenza, which is known to have substantial morbidity and mortality.^1^ A modelling study found that around 28,500 hospitalisations and over 7,100 deaths were attributable to influenza in a mean season in the UK between 1996 and 2009, and the highest incidence rates of hospitalisations and death were found in the elderly population (≥75 years).^2^ As such, efforts to prevent or ameliorate the impact of seasonal influenza have resulted in a well‐established vaccination programme targeting high‐risk populations in the UK, including the elderly population (aged ≥65 years), co‐morbid patients (e.g., chronic respiratory disease) and the immunocompromised.^3^

In contrast, notably less information is published on other seasonal respiratory viruses, such as respiratory syncytial virus (RSV), despite the growing recognition of a potentially considerable disease burden comparable to influenza.^4^ Epidemics of RSV infections usually occur on an annual basis in the UK, following a seasonal pattern that sees a peak in infections recorded around December.^5^ However, in the wake of the coronavirus 2019 (COVID‐19) pandemic, this seasonality has been disrupted and has not yet returned to its pre‐pandemic pattern and thus may have implications for the effectiveness of any seasonal intervention strategy against RSV.^6^, ^7^, ^8^ Most cases present with mild symptoms, such as rhinitis, cough or sometimes fever; however, RSV infections may lead to serious respiratory illness with complications that include pneumonia, exacerbation of chronic obstructive pulmonary disease (COPD) or death.^9^, ^10^, ^11^ Patient groups known to be at higher risk of severe disease include the elderly population, as well as co‐morbid, immunocompromised and co‐infected patient populations.^12^

The burden of RSV is commonly discussed in the context of childhood infections. It was estimated that around 29,160 hospitalisations and 83 deaths among children and adolescents aged 0–17 years were attributable to RSV in the UK in an average season between 1995 and 2009, with the highest proportion of this burden in infants <6 months of age.^13^ However, studies suggest that the burden of RSV in adults may also be substantial.^14^, ^15^ A modelling study for RSV estimated around 17,800 hospitalisations and almost 8,500 deaths were attributable to RSV in an average season in the UK between 1995 and 2009 among adults (≥18 years), with the highest disease burden in the elderly population (93% of deaths in adults occurred in patients ≥65 years).^14^ Another study estimated that RSV accounted for a seasonal annual average of 71 hospitalisations per 100,000 in adults aged 65–74 years and 251 admissions per 100,000 in adults aged ≥75 years between 2010 and 2017 in England.^15^

Despite the burden of RSV highlighted above, there are currently very few therapies available in the UK. Only two anti‐viral therapies are approved and available for children: ribavirin and palivizumab.^16^, ^17^, ^18^, ^19^, ^20^, ^21^ In addition, nirsevimab has recently been approved by the Medicines and Healthcare products Regulatory Agency (MHRA).^22^ No approved therapies are available for UK adults, and only one vaccine (Arexvy) has been recently approved by MHRA for adults aged 60 years or older^23^; however, several promising therapies and vaccines are currently in development.^24^, ^25^, ^26^, ^27^, ^28^ Thus, understanding the burden of RSV in adult populations will be critical to appraise the upcoming therapeutic and preventative modalities.

Several recently published, multi‐national systematic literature reviews (SLRs) have highlighted the appreciable burden of RSV in older and high‐risk adults; however, these studies did not mainly focus on a UK adult population.^29^, ^30^, ^31^ As such, there still remains a need to strengthen the current evidence base on the burden of RSV, particularly in UK adults, to adequately inform assessment of new vaccines and treatments in the UK. Thus, the present SLR set out to characterise the clinical, economic and epidemiological burden of RSV in UK adults, including those at higher risk of severe disease, and to conduct a gap analysis to highlight areas that would benefit from further research.

## 2 METHODS

This SLR was performed in accordance with a pre‐specified protocol, although this was not registered, and reported in line with the Preferred Reporting Items for Systematic Reviews and Meta‐analyses (PRISMA) guidelines.^32^

### 2.1 Search strategy

A de novo SLR was conducted in October 2021, with an SLR update performed in August 2022. Electronic databases (MEDLINE, Embase, Database of Abstracts of Reviews of Effect [DARE], Cochrane Central Register of Controlled Trial [CENTRAL], Cochrane Database of Systematic Reviews [CDSR], National Health Service Economic Evaluation Database [NHS EED] and the International Health Technology Assessment [HTA] database [INAHTA]) were searched from database inception to 7 October 2021 in the original SLR and 8 August 2022 in the SLR update, using search terms for RSV combined with a validated UK geographical filter.^33^ Additionally, conference proceedings of relevant congresses that had taken place between 2019 and 2021 in the original SLR, and any conducted since the original SLR in the SLR update (2022), were screened for relevant research studies, and economic/HTA websites were manually hand‐searched for relevant records. Bibliography searching of identified SLRs, network meta‐analyses, economic evaluations and HTAs was also carried out. The full search strategy is described in Appendix S1 (Tables S1–S9).

### 2.2 Study selection

The eligibility criteria in Table 1 were used to identify studies relevant to this review. The population of interest comprised adults (≥15 years) with RSV infection, including, but not limited to, the high‐risk subgroups analysed in this SLR (elderly population [≥65 years], patients with co‐morbidities or co‐infection and immunocompromised patients). The outcomes of interest included epidemiological, economic and clinical burden outcomes. The studies included in this SLR were observational studies, and no intervention or comparator limits were applied. Due to the aims of this review, only studies conducted in the UK were included, and for this reason, it was considered acceptable to only review publications in the English language. The date limits applied were 2011 for journal articles and 2019 for conference abstracts, in order to only include information relevant to the current disease and treatment landscape.

**TABLE 1:** Eligibility criteria for the SLR.

Domain	Inclusion criteria	Exclusion criteria
Patient population	Adults (≥15 years) with RSV, including but not limited to the following subgroups: Patients with co‐morbidities (e.g., COPD and cardiovascular disease) Immunocompromised patients (e.g., those having undergone HSCT) Elderly adults (≥65 years) Patients with co‐infection (e.g., influenza and COVID‐19)	Paediatric patients (classified as patients aged <15 years) Patients that do not have RSV
Intervention/comparator	Any or none	N/A
Outcomes (*reported for each patient subpopulation of interest to this review*)	Epidemiological outcomes, including: Incidence or prevalence of RSV Incidence or prevalence of respiratory complications of RSV (e.g., pneumonia, exacerbation of COPD and asthma) RSV‐attributable mortality Economic outcomes, including: Direct healthcare costs and resource use (e.g., GP visits, outpatient visits, A&E visits, hospitalisations and length of stay, ICU admissions and length of stay, CCU admissions and length of stay, antibiotic prescriptions, O_2_ and feeding supplementation, readmissions, ventilator use, and long‐term costs) Indirect costs (e.g., productivity, absenteeism) Clinical burden, including: Signs and symptoms (grouped by disease severity, e.g., symptomatic, or severe disease^a^)	Studies not reporting any of the outcomes listed of relevance Studies reporting relevant clinical or economic outcomes, but in groups of a mixed population, without reporting data specifically for the RSV patient group
Study design	Observational studies SLRs, economic evaluations, and HTAs [to be excluded at full text stage after reference list searches]	Interventional studies Non‐systematic reviews Case reports and case studies
Other considerations	Studies conducted in the UK Studies published since 2011 Conference abstracts published in or after 2019 Studies on human subjects Studies published in the English language	Studies in animals In vitro studies in cells, cell lines and/or tissue samples Abstract not in the English language Studies conducted outside the UK Studies published before 2011 Conference abstracts published earlier than 2019

Abbreviations: A&E, accident and emergency; CCU, cardiac care unit; COPD, chronic obstructive pulmonary disease; COVID‐19, coronavirus disease 2019; GP, general practice; HSCT, haematopoietic stem cell transplant; HTA, health technology assessment; ICU, intensive care unit; N/A, not applicable; O2, oxygen; RSV, respiratory syncytial virus; SLR, systematic literature review; TCU, transitional care unit; UK, United Kingdom.

^a^ Severe disease to be classified as RSV requiring hospitalisation or resulting in death; symptomatic disease to be classified as RSV showing signs/symptoms, including RSV resulting in healthcare resource use e.g., GP appointments, or treatment.

The titles and abstracts of publications identified from the database searches were reviewed by two independent reviewers against the eligibility criteria. Where the applicability of the inclusion criteria was unclear, the article was included for full‐text screening. The full‐text publication of each record deemed eligible (or unclear) was further reviewed against the eligibility criteria by two independent reviewers. In cases where insufficient information was provided to adequately assess eligibility, the publication was excluded at the full‐text stage. Hand searching of conference proceedings, economic/HTA websites and bibliographies of the identified SLRs were conducted by a single reviewer with decisions regarding eligibility checked by a second reviewer. Any disagreements were resolved by discussion until a consensus was met. If necessary, a third independent reviewer made the final decision. Full lists of studies included in the SLR and excluded at the full‐text stage review, including the reasons for exclusion, are provided in Appendix S2 (Table S10) and Appendix S3 (Tables S11–S12), respectively.

Key information from all included studies was extracted into a pre‐specified data extraction grid in Microsoft Excel; extracted information included characteristics of the patient population, study characteristics and the relevant outcome data presented. Data extraction was conducted by a single reviewer, and data were independently verified by a second reviewer. Where relevant, graphical data digitisation was performed using Digitizelt software; digitisation was not performed for continuous data.

A gap analysis was additionally conducted to identify subgroups of interest and burden outcomes where little or no data exist in the literature. The analysis was completed by one individual and independently verified by another individual.

### 2.3 Risk of bias

Quality assessments of included studies were also conducted using the quality assessment tool developed by The Alberta Heritage Foundation for Medical Research (AHFMR).^34^ Study quality assessment was conducted by a single reviewer and independently verified by a second reviewer.

## 3 RESULTS

### 3.1 Search results

In the original SLR, a total of 2,507 records were retrieved by the electronic database searches, and an additional 635 were captured by supplementary searches of conference abstracts and economic/HTA websites. Of these, 22 publications reporting on 22 studies were found to fulfil the eligibility criteria for inclusion in this review.

In the SLR update, a total of 253 novel records were retrieved by the electronic database searches and a further 327 captured by supplementary searches of conference abstracts and economic/HTA websites. Of these, six publications reporting on six studies were found to fulfil the eligibility criteria for inclusion in the review.

**FIGURE 1:**
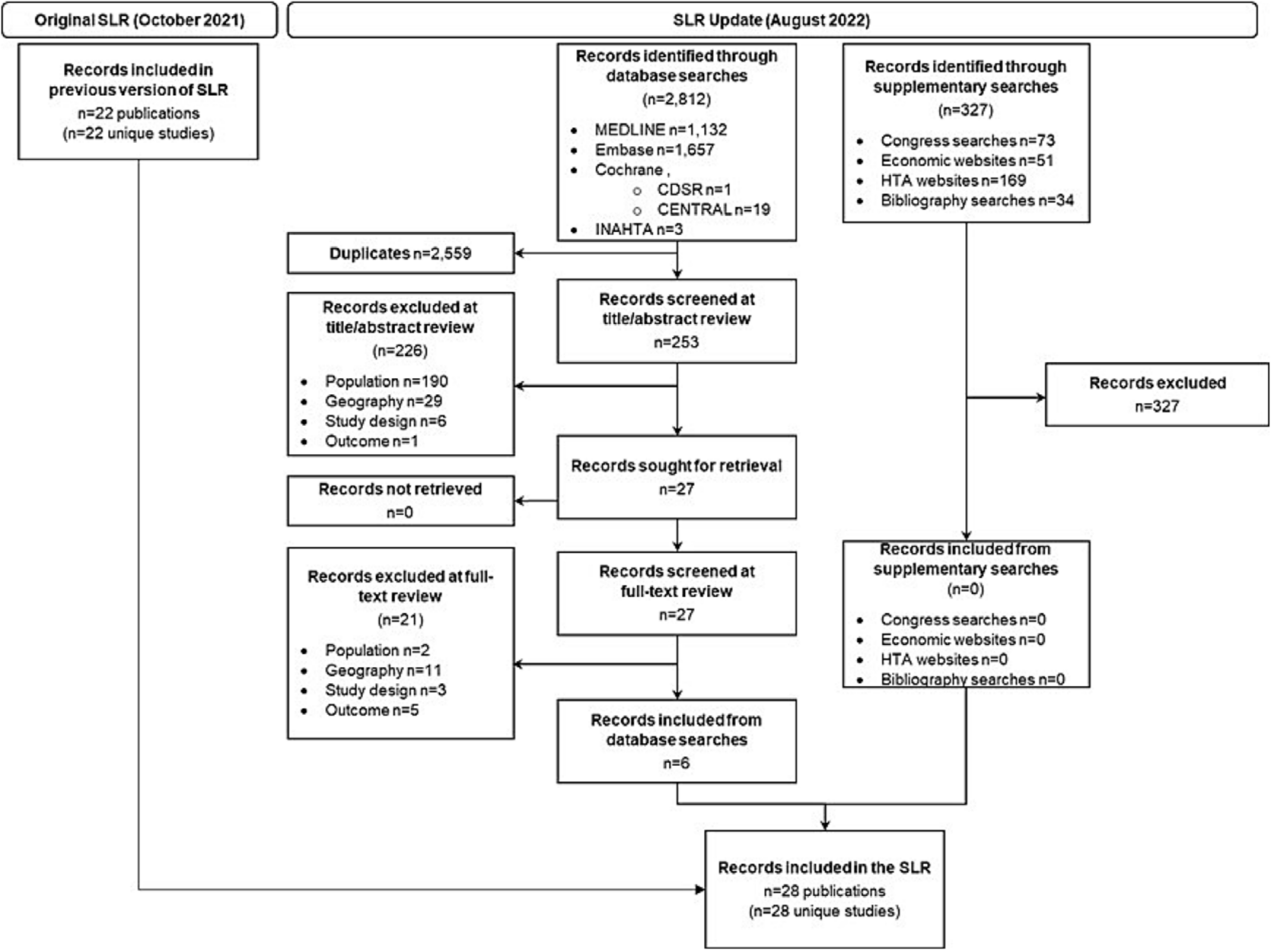
PRISMA diagram for the original SLR and SLR update. Abbreviations: CDSR, Cochrane Database of Systematic Reviews; CENTRAL, Cochrane Central Register of Controlled Trials; CRD, Centre for Reviews and Dissemination; DARE, Database of Abstracts of Reviews of Effects; HTA, Health Technology Assessment; INAHTA, International Health Technology Assessment Database; NHS EED, National Health Service Economic Evaluation Database; PRISMA, Preferred Reporting Items for Systematic Reviews and Meta‐Analyses.

In total, 28 studies were identified in the original SLR and SLR update. The PRISMA diagram for the SLR update is provided in Figure 1 and for the original SLR in Appendix S4.

Among the 28 publications reporting on 28 unique studies that were included in the SLR, 25 studies reported epidemiological data,^14^, ^35^, ^36^, ^37^, ^38^, ^39^, ^40^, ^41^, ^42^, ^43^, ^44^, ^45^, ^46^, ^47^, ^48^, ^49^, ^50^, ^51^, ^52^, ^53^, ^54^, ^55^, ^56^, ^57^, ^58^ nine studies reported economic data^14^, ^15^, ^35^, ^41^, ^45^, ^54^, ^55^, ^57^, ^59^, ^60^ and two studies reported clinical data.^45^, ^48^ The most commonly reported epidemiological outcome was incidence of RSV infection, reported by 24 studies in total,^35^, ^36^, ^37^, ^38^, ^39^, ^40^, ^41^, ^42^, ^43^, ^44^, ^45^, ^46^, ^47^, ^48^, ^49^, ^50^, ^51^, ^52^, ^53^, ^54^, ^55^, ^56^, ^57^, ^58^ followed by mortality, reported by five studies.^14^, ^35^, ^41^, ^45^, ^57^ None of the studies reported data regarding the epidemiology of respiratory complications of RSV. Economic outcomes included only direct resource use under both inpatient and outpatient settings and no studies reported any indirect resource use or disease‐related costs. Clinical outcomes reported by two studies included presence of symptoms and/or their degree of severity measured by symptom scores.^45^, ^48^

### 3.2 Burden of RSV in a general population and/or with a respiratory indicator

Seventeen studies reported on a general population and/or a population with a respiratory indicator, defined as patients assumed to be displaying infectious respiratory infection symptoms, having undergone RSV testing in a healthcare setting; their characteristics and extracted outcomes are described in Appendix S5 (Table S13).^14^, ^36^, ^42^, ^43^, ^44^, ^46^, ^47^, ^48^, ^49^, ^51^, ^52^, ^53^, ^54^, ^55^, ^56^, ^59^, ^60^

Epidemiological outcomes were reported by 15 studies; 14 reported on RSV infection incidence,^36^, ^42^, ^43^, ^44^, ^46^, ^47^, ^48^, ^49^, ^51^, ^52^, ^53^, ^54^, ^55^, ^56^ and 1 study reported mortality.^14^

**FIGURE 2:**
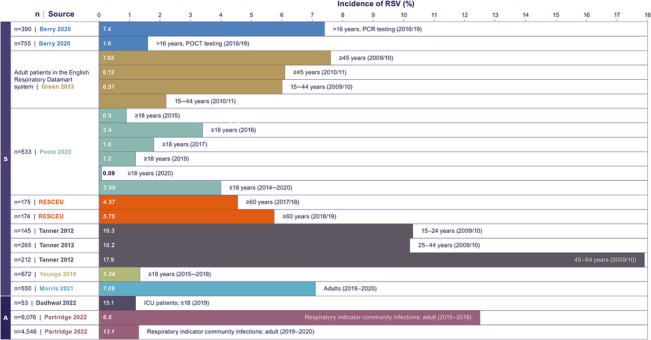
Incidence of RSV in patients with a respiratory indicator. Italicised values were manually calculated; data from relevant studies identified in this SLR that only represented RSV infection incidence graphically and/or used units other than percentage^43^, ^44^, ^53^, ^55^ are not included in this figure. Abbreviations: A, annual; ICU, intensive care unit; PCR, polymerase chain reaction; POCT, point‐of‐care testing; S, seasonal.

Eight studies reported seasonal RSV infection incidence (defined as incidence with data collection restricted to the RSV season) for patients with a respiratory indicator.^36^, ^42^, ^44^, ^46^, ^47^, ^49^, ^51^, ^52^ One of these studies only represented RSV infection incidence data graphically and therefore was not included in the further summary of incidence values.^44^ In the other seven studies, seasonal incidence ranged widely from 0.09% (patients ≥18 years, 2020^49^; hospital setting) to 17.9% (patients 45–64, 2009/10^51^; general practitioner [GP]/hospital setting) (Figure 2).^49^, ^51^

Five studies reported on annual (continuous) RSV infection incidence (defined as data collected continuously over a year or more) within patients with a respiratory indicator.^43^, ^53^, ^54^, ^55^, ^56^ One of these studies only represented RSV infection incidence data graphically and therefore was not included in the further summary of incidence values.^53^ Two studies clearly reported RSV infection rates as cumulative incidence across two settings; intensive care unit (ICU) patients and patients attending a hospital with respiratory indicators.^54^, ^56^ In these studies, the annual incidence ranged between 6.6% (adult patients, 2015–2018^56^; hospital) and 15.1% (patients ≥18 years, 2019^54^; ICU).^54^, ^56^ The two other studies reported modelled RSV incidence rates.^43^, ^55^ One reported RSV incidence rate as 0.1 per 1,000 people per year (patients 18–64^55^; hospital), and the other reported RSV incidence to range between 0.784 and 2.42 positive infections per 100,000 samples, with incidence rate increasing by age (0.784, patients 15–24; 1.19, patients 25–44; 2.42, patients 45–64^43^; hospital).

Only one study was identified reporting RSV infection incidence in a general cohort. Ponsford et al.^48^ compared healthy controls with immunocompromised patients with primary antibody deficiency. Among healthy controls, the study found a very low measured annual RSV infection incidence of 0.3% (*n* = 38; number of swabs taken *n* = 626).

One study reported estimated RSV‐attributable mortality in a respiratory indicator cohort, assessing adult patients diagnosed with RSV in the UK‐wide Office for National Statistics database.^14^ The study found a total of 8,482 deaths per season were attributable to RSV between 1995 and 2009. Among all deaths attributable to respiratory disease, 4.2% and 5.9% for the 18–49 and 50–64 age group, respectively, were estimated to be attributable to RSV.

Clinical burden evidence was identified in only one study,^48^ which reported the Burden of Infection as measured by Primary Antibody Deficiency (BIPAD‐Q) symptom score in primary antibody deficiency (immunocompromised) patients with RSV (*n* = 41) and otherwise healthy controls with RSV (*n* = 38). The scale ranged from 0 (*asymptomatic*) to 7 (*multiple, severe symptoms*). In the healthy control group, the symptom score decreased by 0.143 in the 7‐day period following RSV detection, compared with an increase of 0.469 in the primary antibody deficient group.

Four studies that reported economic burden outcomes in a general population and/or patients with a respiratory indicator reported direct resource use across various settings of care, including GP visits, inpatient hospital stays and telehealth calls (see Appendix S5 for results).^14^, ^54^, ^55^, ^59^, ^60^

Of the studies assessing patients with a respiratory indicator, two reported GP visits, with one estimating the mean number of weekly GP consultations attributed to RSV between 2011 and 2015.^14^, ^59^ Morbey et al.^59^ found 2.18 weekly GP consultations in adults aged 15–64 years were attributed to RSV in a modelled cohort of 10,000 patients (6,600 among them aged 15–64 years) in England during a winter season (Weeks 40 to 20). Fleming et al.^14^ reported the mean number of GP episodes per average season attributable to RSV across the UK as 487,247 within patients ≥18 years old.

### 3.3 Burden of RSV in elderly patients

Among the high‐risk subpopulations, the largest body of UK evidence was found for the elderly population (≥65 years). The elderly are known to be at a higher risk of serious illness from RSV and other respiratory viruses, owing to higher rates of co‐morbidities and lower immunity when compared with a younger patient population.^61^

Ten studies identified in this SLR reported on an elderly cohort (aged ≥65 years).^14^, ^15^, ^37^, ^38^, ^43^, ^51^, ^53^, ^55^, ^59^, ^60^ Characteristics of these studies and extracted outcomes are described in Appendix S5 (Table S14).

Seven studies reported on epidemiological burden in the elderly.^14^, ^37^, ^38^, ^43^, ^51^, ^53^, ^55^ Of these six studies captured RSV incidence in patients ≥65 years^37^, ^38^, ^43^, ^51^, ^53^, ^55^ and two studies in patients ≥75 years.^37^, ^55^

**FIGURE 3:**
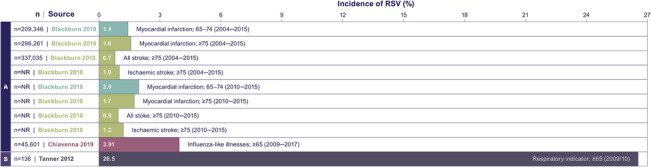
Incidence of RSV in elderly patients. Data from relevant studies identified in this SLR that only represented RSV infection incidence graphically and/or used units other than percentage^43^, ^44^, ^53^, ^55^ are not included in this figure. Abbreviations: A, annual; NR, not reported; RSV, respiratory syncytial virus; S, seasonal.

A seasonal RSV incidence of 26.5% was reported in one study that collected data from hospitals and GPs in the West Midlands region of England during the 2009/2010 influenza pandemic (≥65 years).^51^ Other studies reported on annual incidence rates in this population, which were considerably lower. The average annual incidence in patients ≥65 years ranged between 1.4% (myocardial infarction [MI] patients, 65–74 years^37^; hospital settings) and 3.91% (≥65 years^38^; GP settings) (Figure 3). In the former, RSV incidence was reported in elderly patients with co‐morbidity, where in the latter, incidence was reported in patients presenting with influenza‐like illness.^37^, ^38^

Five studies captured in the SLR assessed the incidence of RSV across different age groups, with all finding the incidence of RSV increasing with older age.^38^, ^42^, ^43^, ^51^, ^55^ For example, Johannesen et al.^55^ estimated incidence rates per 1,000 patients in England aged between 18–64 (0.1), 65–74 (0.9), 75–84 (2.8) and ≥85 years (6.0).

One study reporting modelled annual (continuous) incidence rates in patients aged ≥75 years with RSV following MI and stroke admission found incidence rates to be lowest in patients with stroke (0.7%) and the highest in patient presenting with MI (1.7%).^37^ Another study modelling annual (continuous) incidence rates per 1,000 patients found rates to be highest in the 75–84 (England: 2.8; Scotland: 3.0) and ≥85 (England: 6.0; Scotland: 5.0) age groups compared with the 65–74 group (England: 0.9; Scotland: 0.6).^55^

One study presented data for a cohort with a respiratory indicator from both GP and hospital settings, finding 93% of all deaths attributable to RSV occurred in elderly patients (≥65 years old).^14^

No studies reflecting the clinical burden of RSV in the elderly population were found in this SLR.

All five of the studies reporting economic burden outcomes in an elderly population reported direct resource use.^14^, ^15^, ^55^, ^59^, ^60^ Morbey et al.^59^ found 0.771 weekly RSV‐attributable GP consultations in a modelled cohort of 10,000 patients (1,710 of them aged ≥65 years), in England during a winter season (Weeks 40 to 20). Fleming et al.^14^ reported the mean number of GP episodes per average season attributable to RSV across the UK as 175,070 in patients ≥65 years, compared with 487,247 in all adult patients (≥18 years). Additionally, the mean seasonal GP visits per 100,000 patients was reported to be higher in the ≥75 age group (2,175) compared with the 65–74 age group (1,742).

Sharp et al.^15^ and Fleming et al.^14^ reported similar and substantial mean seasonal rates of hospital admissions per 100,000 patients in the elderly population (≥65 years), with higher rates in the ≥75 age group (251 and 234, respectively) versus the 65–74 age group (71 and 86, respectively). Johannesen et al.^55^ also reported hospital admission data, finding the proportion of average yearly respiratory admissions associated with RSV ranged from 2.4% (18–64 years, England) to 7.2% (75–84 years, England). No evidence for any indirect resource use or disease‐related costs were reported by any of the studies.

### 3.4 Burden of RSV in other high‐risk patient groups

Other high‐risk groups investigated in this review included co‐morbid, immunocompromised and co‐infected patient populations, as they are known to be at a higher risk of morbidity from respiratory infections.^12^ Characteristics of the studies reporting on these patient subgroups, as well as extracted outcomes for all studies, are provided in Appendix S5 (Tables S15–S17).

**FIGURE 4:**
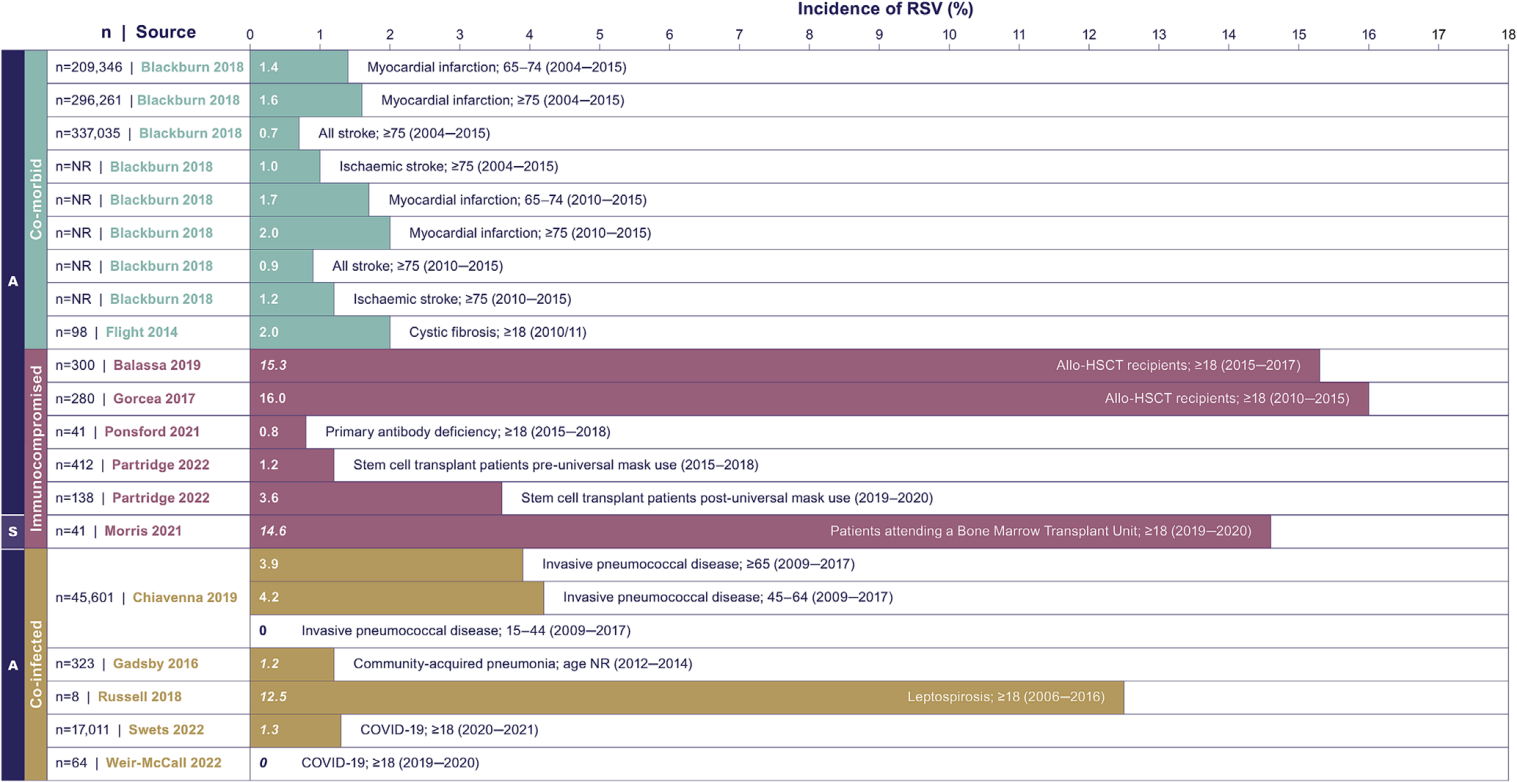
Incidence of RSV in other high‐risk groups. Italicised values were manually calculated; all data presented were collected as annual testing except for Morris et al.,^47^ which presented seasonal testing. Abbreviations: COVID‐19, coronavirus‐19; HSCT, haematopoietic stem‐cell transplantation; NR, not reported; RSV, respiratory syncytial virus.

For the co‐morbid patient population, the evidence was highly limited, with only two studies identified.^37^, ^39^ Both studies in a hospital setting reported an annual RSV infection incidence of 0.7% (≥75 years, stroke patients^37^) and 2% (≥18 years, cystic fibrosis patients^39^) (Figure 4). No discernible trends were observed across the results reported for co‐morbid patient populations, based on the limited evidence identified.

Six studies reported on RSV infection incidence in immunocompromised patients.^35^, ^41^, ^45^, ^47^, ^48^, ^56^ A wide range of RSV infection incidence rates (seasonal and annual) were reported (0.8–16%) (Figure 4).^41^, ^47^, ^56^ Four of the studies reporting on immunocompromised patient populations discussed RSV symptom presentation or symptom scores.^35^, ^41^, ^45^, ^48^ As previously detailed, Ponsford et al.^48^ found that the immunocompromised cohort (*n* = 41) had a higher symptom load 7 days after RSV detection compared with the healthy controls with RSV (*n* = 38). Two other studies compared the incidence of upper and lower respiratory tract infections (URTIs and LRTIs) in RSV patients undergoing allogeneic haematopoietic stem cell transplantation (HSCT). Balassa et al.^35^ reported a slightly higher rate of LRTIs compared with URTIs between November 2015 to December 2017 (53.1% vs. 46.9%; *n* = 46), whereas Gorcea et al.^41^ observed a higher rate of URTIs between December 2010 and February 2015 (69.6% vs. 30.4%; *n* = 23). Three studies reported on the economic burden of RSV in the immunocompromised. All three studies reported drug usage and two reported inpatient stays, finding moderate resource use.^35^, ^41^, ^45^

Five studies reported on a co‐infected patient population and presented the annual incidence of RSV in patients with invasive pneumococcal disease, leptospirosis, COVID‐19 and community‐acquired pneumonia.^38^, ^40^, ^50^, ^57^, ^58^ Incidence rates across these studies ranged from 0% (15–44 years, invasive pneumococcal disease; laboratory and database setting^38^ and >18 years, COVID‐19; national extracorporeal membrane oxygenation service centres^58^) to 12.5% (≥18 years, leptospirosis, tertiary care setting^50^) (Figure 4). One study that reported direct resource use (ventilator use, steroid use and ICU admissions) in RSV and COVID‐19 co‐infected patients reported that most patients (72%) required treatment with systemic corticosteroids; critical care admissions were required in 23.4% of patients and ventilator use in 16.8% of patients.^57^ However, the resource use in RSV infection compared to COVID‐19 mono‐infection was either not analysed or not significantly different. No further economic evidence and no clinical burden evidence was identified for this patient subgroup.

### 3.5 Gap analysis

The SLR identified a notable paucity of information on the burden of RSV in UK adults. The outcomes reported in all captured studies are summarised in Table 2. In order to explore the current evidence gaps systematically, a gap analysis framework was used, highlighting the need for further research on the burden of RSV in different adult populations (a summary of the gap analysis results is presented in Table 3; the full results can be found in Appendix S6).

**TABLE 2:** Summary of outcomes reported by publication.

Outcome		Balassa 2019^35^	Berry 2020^36^	Blackburn 2018^37^	Chiavenna 2019^38^	Dadhwal 2022^54^	Fleming 2015^14^	Flight 2014^39^	Gadsby 2016^40^	Gorcea 2017^41^	Green 2013^42^	Hodgson 2020^43^	Hughes 2016^44^	Inkster 2017^45^	Johannesen 2022^55^	RESCEU^46^	Morbey 2017^60^	Morbey 2018^59^	Morris 2021^47^	Partridge 2022^56^	Ponsford 2021^48^	Poole 2020^49^	Russell 2018^50^	Sharp 2022^15^	Swets 2022^57^	Tanner 2012^51^	Weir‐Mccall 2022^58^	Youngs 2019^52^	Zhao 2014^53^
Economic	GP visits						Y											Y											
	Drug use	Y					Y			Y				Y											Y				
	NHS 111 calls																Y												
	Inpatient stays^a^	Y					Y			Y					Y									Y	Y				
	Outpatient treatment	Y																											
	ICU admission	Y								Y															Y				
	Ventilator use	Y																							Y				
Epidemiology	Incidence	Y	Y	Y	Y	Y		Y	Y	Y	Y	Y	Y	Y	Y	Y			Y	Y	Y	Y	Y		Y	Y	Y	Y	Y
	Mortality	Y					Y			Y				Y											Y				
	Presentation of RSV	Y								Y																			
Clinical	Presence of symptoms^b^													Y															
	Symptom score																				Y								
	Specific symptoms^c^													Y															

Abbreviations: GP, general practice; ICU, intensive care unit; NHS, National Health Service; RSV, respiratory syncytial virus.

^a^ Inpatient stays include both hospital admission data and length of hospital stay.

^b^ Presence of symptoms refers to proportion of patients who display any symptoms of RSV.

^c^ Specific symptoms refer to the proportion of patients who display certain RSV symptoms (this included cough, weakness, shortness of breath, sputum, wheezing, fever, sore throat, headache, chest pain, vomiting, fatigue and myalgia).

**TABLE 3:**
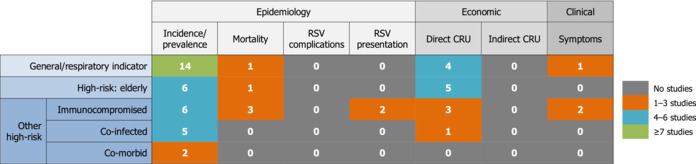
Gap analysis of included studies.

Abbreviations: CRU, cost and resource use; RSV, respiratory syncytial virus.

### 3.6 Quality assessment

A summary of the AHFMR quality assessment checklist results conducted for each study included in the SLR is detailed in Appendix S7 (Table S18). Overall, most included studies (23/28) were considered to have a low risk of bias. A total of five studies had a high risk of bias, measured as reporting high risk for 3 or more domains in the AHFMR checklist.^43^, ^45^, ^53^, ^59^, ^60^

## 4 DISCUSSION

The data found in this SLR on the disease burden of RSV in the UK adult population are highly heterogenous. As such, comparisons among studies should be interpreted with caution given several factors may have affected the estimates: methodological differences across studies, annual variations in RSV activity, data collection periods (seasonal versus annual), patient sampling methods, RSV testing methods, healthcare seeking behaviour of the underlying population and the impact of COVID‐19 on the altered seasonality of RSV. In addition, most studies in this SLR did not adjust for confounding, further complicating potential comparisons (Appendix S7). Due to these limitations, it was not feasible to conduct a meta‐analysis of the studies included in this SLR.

Seasonal RSV infection incidence in patients with a respiratory indicator ranged between 0.09% and 17.9%, while annual incidence was between 6.6% and 15.1%.^49^, ^51^, ^54^, ^56^ The results showed no discernible trend relating to study setting or RSV detection method. However, variation in RSV infection incidence according to year of data collection was noted, which could be explained by variations in RSV incidence and/or testing behaviour. For instance, the lowest seasonal incidence (0.09%, patients ≥18 years, Poole et al.^49^) was likely due to the social distancing measures imposed during the COVID‐19 pandemic. The highest RSV infection incidence was reported during the 2009–2010 influenza pandemic, likely due to the increased testing behaviour for respiratory infections.^51^

Generally, RSV is believed to be underdiagnosed, especially in milder cases, due to low testing rates and non‐specific symptoms, as well as the difficulty in adult diagnosis compared with children, due to insensitivity of diagnostic tests caused by a lower viral load in nasal secretions.^62^ Consequently, the higher seasonal incidence rates detected during the influenza pandemic may be hypothesised to be closer to the real RSV incidence.^9^, ^12^, ^62^, ^63^ Interestingly, on the other hand, Blackburn et al.^37^ assessed the impact of the 2009 influenza pandemic on RSV testing behaviours, comparing the incidence of RSV detected from 2004–2015 and 2010–2015, and demonstrated similar RSV detection over these two periods, suggesting that no significant change occurred to viral testing behaviour as a result of the influenza pandemic. The growing awareness of the burden of RSV, as well as the advances in respiratory virus detection brought forward by the COVID‐19 pandemic,^64^ may potentially lead to improved RSV testing rates in the near future, providing a more accurate epidemiological picture of RSV.

The economic outcomes captured suggest RSV may place a high economic burden on the healthcare system, with the studies captured in this SLR reporting a range of resource use associated with infection, including hospital admissions, GP visits, drug use and telehealth services, though this requires verification by future research. Of particular note, Fleming et al.^14^ suggested that RSV may place a similar economic burden on the NHS as influenza. The study estimated that in most seasons between 2001 and 2009, more GP visits and hospitalisations were attributable to RSV in adults than influenza. Sharp et al.^15^ similarly estimated a high hospitalisation burden associated with RSV in the elderly (≥65 years) in England. Further, Morbey et al.^59^ found 2.18 weekly consultations on average were attributable to RSV in adults aged 15–64 in a modelled cohort of 10,000 patients in the 2013/2014 and 2014/2015 RSV seasons compared with 3.58 weekly consultations associated with influenza. Despite implying that RSV has a slightly lower burden on the healthcare system than influenza, this still highlights the high number of GP episodes attributable to RSV in adults and consequently the high economic burden of RSV.^14^, ^59^ It is worth noting that studies conducted in other countries report conflicting findings, highlighting the need to further collect country‐specific data on RSV burden and to interpret the data with caution.^65^, ^66^, ^67^ In addition, the captured studies discussing the economic burden of RSV focused on direct resource use, while information on costs and indirect resource use was not provided.

Among the high‐risk populations, the largest body of evidence was found for the elderly patients. Similar to the general population, the incidence of RSV infections found in this sub‐population was highly variable, with one study reporting the seasonal incidence of RSV infections in the West Midlands region of England in a hospital and GP setting among the patients ≥65 years as high as 26.5%,^51^ and the annual incidence reported in other studies being considerably lower.^37^, ^38^ This heterogeneity demonstrates that the measured incidence is linked to the data collection period (annual vs. seasonal, as well as the year of data collection) and the specific patient population. Five studies captured in this SLR reported incidence data across different age groups and all found RSV infection incidence rates to increase across the age groups assessed with the highest rate reported in the elderly patient group (≥65 years). Importantly, the mortality in the elderly group was found to be substantial. One study found that for a cohort with a respiratory indicator from both GP and hospital settings, 8,482 deaths per season in adults (≥18 years) in the UK were attributed to RSV, and 93% of these deaths occurred in elderly patients (≥65 years); this mortality burden is substantial and comparable to that of influenza.^2^, ^14^

A number of studies were found reporting the burden of RSV in other high‐risk groups, which in this SLR were defined as co‐morbid, immunocompromised and co‐infected patient populations. No discernible trends were observed across the results reported for co‐morbid patient populations, based on the limited evidence identified.

For the immunocompromised patient population, a wide range of RSV infection incidence rates (seasonal and annual) were reported, ranging from 0.8% to 16%, suggesting that in some cases these patients might be more susceptible to RSV infection.^41^, ^47^, ^56^ These results concur with the findings of a published SLR considering studies across global geographies, which found an incidence range of 2.1% to 19.6% in HSCT patients.^68^ The results of our SLR also suggest that immunocompromised patients may experience a higher symptom burden compared to healthy individuals, as well as present a substantial economic burden.^48^, ^69^

Similar to other patient groups, the identified evidence for co‐infected patients was found to be heterogenous, which in some cases may be explained by a small sample size.^50^

The gap analysis highlighted that only a small number of studies, if any, report economic, epidemiological and/or clinical burden outcomes for each patient population of interest in this review. The lack of information was particularly notable for the clinical burden data and indirect economic data. Most studies captured are non‐comparable, due to heterogeneity in study design, or differences in the patient group eligibility criteria. Due to the paucity of evidence identified in this SLR, it is difficult to fully interpret the public health and economic impact of RSV infections in the UK. Future nationally representative database analyses are required to fully assess the economic burden of RSV, and large, multicentre registry analyses are required to characterise the clinical burden of RSV.

This SLR was conducted in line with the Cochrane Handbook for Systematic Reviews of Interventions.^70^ A potential limitation of this review is that studies included were limited to being published in 2011 or later; however, this date limit was selected to ensure that only the data relevant to the current RSV landscape were identified. A further limitation is that the database search strategy was developed to capture studies that reported ‘RSV’ or ‘respiratory syncytial virus’ within the abstract, title or keywords. It is possible that studies assessing other viruses might have reported relevant burden data for RSV as a comparison within the full text and not specified this within the abstract. This limitation however was unavoidable, in order to ensure a manageable number of records were captured from the database searches. Another limitation of this SLR is that it included estimated/modelled data alongside more robust measured data, which may weaken any of the conclusions that incorporate the modelled data.

## 5 CONCLUSION

The data collected in this SLR show that the burden of RSV could be substantial and comparable to that of influenza, especially in high‐risk populations. However, the available evidence is highly heterogenous, which precludes meta‐analysis of the discovered data, and a high number of missed areas was identified by the gap analysis. There is clear need for further studies of RSV burden in the UK, particularly in light of RSV treatments and vaccines currently undergoing development, as well as advancements in diagnostics, in order to inform their future applications.

## AUTHOR CONTRIBUTIONS

**Tom Wilkinson:** Conceptualization; visualization; writing—original draft; writing—review and editing. **Steph Beaver:** Conceptualization; data curation; formal analysis; investigation; methodology; project administration; software; supervision; validation; visualization; writing—original draft; writing—review and editing. **Malcolm Macartney:** Conceptualization; data curation; formal analysis; funding acquisition; project administration; resources; supervision; visualization; writing—original draft; writing—review and editing. **Eve McArthur:** Conceptualization; data curation; formal analysis; investigation; methodology; project administration; software; supervision; validation; visualization; writing—original draft; writing—review and editing. **Vaishali Yadav:** Conceptualization; data curation; formal analysis; investigation; methodology; project administration; software; supervision; validation; visualization; writing—original draft; writing—review and editing. **Annie Lied‐Lied:** Conceptualization; data curation; formal analysis; funding acquisition; project administration; resources; supervision; visualization; writing—original draft; writing—review and editing.

## CONFLICT OF INTEREST STATEMENT

Tom Wilkinson received grant funding and consultancy fees from AstraZeneca, BerGenBio, Gilead, Janssen, My mHealth, Sanofi, Synairgen, Teva and UCB and grant funding from Asthma + Lung UK, Medical Research Council and National Institute for Health and Care Research; Steph Beaver, Eve McArthur, and Vaishali Yadav are employees of Costello Medical Ltd; Malcolm Macartney and Annie Lied‐Lied are shareholders of Johnson & Johnson and employees of Janssen‐Cilag Ltd.

6

### PEER REVIEW

The peer review history for this article is available at https://www.webofscience.com/api/gateway/wos/peer-review/10.1111/irv.13188.

## DATA SHARING STATEMENT

All data relating to this manuscript are available within the manuscript or the supporting information.

## Supporting information


**Appendix S1.** Search Strategy.
**Appendix S2.** Full List of Studies Included in the SLR.
**Appendix S3.** Full Lists of Studies Excluded at Full‐Text Review Stage.
**Appendix S4.** PRISMA Diagram for the Original SLR Search.
**Appendix S5.** Characteristics of Studies Included in the SLR and Extracted Results.
**Appendix S6.** Gap Analysis of Studies Included in the SLR.
**Appendix S7.** Quality Assessment of Studies Included in the SLR.

**Data S1.** PRISMA 2020 Checklist.
