# Supplementary material for: Burden of respiratory syncytial virus in adults in the United Kingdom: A systematic literature review and gap analysis
Source: Influenza Other Respir Viruses. 2023 Sep 21;17(9):e13188. doi: 10.1111/irv.13188 (PMC10511839; doi:10.1111/irv.13188)
Supplement: Supplementary file 1 — Appendix S1. Search Strategy. Appendix S2. Full List of Studies Included in the SLR. Appendix S3. Full Lists of Studies Excluded at Full‐Text Review Stage. Appendix S4. PRISMA Diagram for the Original SLR Search. Appendix S5. Characteristics of Studies Included in the SLR and Extracted Results. Appendix S6. Gap Analysis of Studies Included in the SLR. Appendix S7. Quality Assessment of Studies Included in the SLR. [file IRV-17-e13188-s002.docx]

**SUPPLEMENTARY APPENDIX**

Contents

[List of Supplementary Tables 2](#_Toc143776258)

[Appendix S1. Search Strategy 3](#_Toc143776259)

[Appendix S2. Full List of Studies Included in the SLR 18](#_Toc143776266)

[Appendix S3. Full Lists of Studies Excluded at Full-Text Review Stage 20](#_Toc143776267)

[Appendix S4. PRISMA Diagram for the Original SLR Search 30](#_Toc143776268)

[Appendix S5. Characteristics of Studies Included in the SLR and Extracted Results 31](#_Toc143776269)

[Appendix S6. Gap Analysis of Studies Included in the SLR 48](#_Toc143776270)

[Appendix S7. Quality Assessment of Studies Included in the SLR 51](#_Toc143776271)

[References 55](#_Toc143776272)

List of Supplementary Tables

[Table S1. Search terms for use in MEDLINE (searched via the Ovid SP platform) 5](#_Toc141705471)

[Table S2. Search terms for use in Embase (searched via the Ovid SP platform) 6](#_Toc141705472)

[Table S3. Search terms for use in the CDSR and CENTRAL databases (searched via the Wiley platform) 8](#_Toc141705473)

[Table S4. Search terms for use in DARE and the NHS-EED (searched via the York CRD platform) 9](#_Toc141705474)

[Table S5. Search terms for the International HTA Database (searched via the INAHTA platform) 10](#_Toc141705475)

[Table S6. Search strategies for hand-searching of relevant congresses in the original SLR and SLR update 12](#_Toc141705476)

[Table S7. Search strategy for HTA website searches 16](#_Toc141705477)

[Table S8. Search strategy for economic website searches in original SLR 17](#_Toc141705478)

[Table S9. Search strategy for economic website searches in SLR update 17](#_Toc141705479)

[Table S10. List of studies included in the SLR 18](#_Toc141705480)

[Table S11. List of studies excluded at the full-text stage review in the original SLR 20](#_Toc141705481)

[Table S12. List of studies excluded at the full-text stage review in the SLR update 27](#_Toc141705482)

[Table S13. Characteristics and reported outcomes of studies reporting RSV burden in a general population and/or with a respiratory indicator 31](#_Toc141705483)

[Table S14. Characteristics and reported outcomes of studies reporting RSV burden in an elderly population 38](#_Toc141705484)

[Table S15. Characteristics and reported outcomes of studies reporting RSV burden in a co-morbid population 42](#_Toc141705485)

[Table S16. Characteristics and reported outcomes of studies reporting RSV burden in an immunocompromised population 43](#_Toc141705486)

[Table S17. Characteristics and reported outcomes of studies reporting RSV burden in a co-infected population 46](#_Toc141705487)

[Table S18. Summary of study quality as assessed by the AHFMR checklist^156^ 52](#_Toc141705488)

Appendix S1. Search Strategy

The original SLR and SLR update were performed in accordance with a pre-specified protocol. This involved searching electronic databases, manual hand searching of key conference proceedings, key Health Technology Assessment (HTA) body websites, health economics databases, and bibliographies of any relevant systematic reviews, (network) meta-analyses ([N]MAs), economic evaluations or HTAs identified during the review.

Electronic Databases

The following databases were searched:

1. MEDLINE(R) Epub Ahead of Print, In-Process & Other Non-Indexed Citations, Ovid MEDLINE(R) Daily and Ovid MEDLINE(R), from 1946 to 7th October 2021 (original SLR) and from 1946 to 8th August 2022 (SLR update)
2. Embase (via Ovid SP), from 1974 to 7th October 2021 (original SLR) and from 1946 to 8th August 2022 (SLR update)
3. The Cochrane Library (via the Wiley Online platform), including:
   1. Cochrane Database of Systematic Reviews (CDSR), Issue 10 of 12, 2021 (original SLR), Issue 8 of 12, 2022 (SLR update)
   2. The Cochrane Central Register of Controlled Trials (CENTRAL), Issue 10 of 12, 2021 (original SLR), Issue 7 of 12, 2022 (SLR update)
4. The University of York Centre for Reviews and Dissemination platform (only searched in the original SLR as the database has not been updated since 2015):
   1. National Health Service Economic Evaluation Database (NHS EED), Issue 2 of 4, April 2015
   2. The Database of Abstracts of Reviews of Effects (DARE), Issue 2 of 5, April 2015
5. The International HTA Database, searched 7th October 2021 (original SLR) and 9th August 2022 (SLR update)

MEDLINE, MEDLINE In-Process, MEDLINE Epub Ahead of Print and Embase were searched separately via the Ovid SP platform. The Cochrane Library databases were searched simultaneously via the Wiley Online platform. The DARE and NHS EED databases were searched simultaneously via the York Centre for Reviews and Dissemination (CRD) platform. The International HTA database was searched via the International Network of Agencies for Health Technology Assessment (INAHTA) platform.

The DARE and NHS EED databases were only searched in the original SLR. The databases were not re-searched in the SLR update as the databases were last updated in April 2015, and are no longer maintained, so would not contain any novel records to review.

During the SLR update, database searches were re-run from database inception and the search results deduplicated against the original SLR EndNote library to ensure any records added to the databases since the date of original search were captured during the update.

Search Terms

The search strategy included free text and subject heading terms grouped into the following categories, and combined, to focus the results of the database searches:

1. RSV: terms for ‘respiratory syncytial virus’ were used to identify studies assessing the population of relevance to this review
2. UK (National Institute for Health and Care Excellence [NICE] filter): terms for ‘United Kingdom’, and specific cities in the UK were used to identify studies assessing relevant geographical locations
3. Exclusion: terms for ‘animal’ studies; irrelevant study designs including single ‘case reports’. The date limits applied were 2011 for journal articles and 2019 for conference abstracts

Search terms were identical for both the original SLR and the SLR update. The search terms used in MEDLINE and Embase are presented in Table S1 and Table S2, respectively. The search terms used in CDSR and CENTRAL are presented in Table S3, and the search terms used in DARE and NHS-EED are presented in Table S4. The search terms for the International HTA database are presented in Table S5. Results from the database searches were downloaded into an EndNote® database and de-duplicated before record screening. Update SLR database results were deduplicated against the original SLR hits.

Table S1. Search terms for use in MEDLINE (searched via the Ovid SP platform)

| **Term Group** | **#** | **Search Terms** | **Original SLR Results (07/10/2021)** | **SLR Update Results (08/08/2022)** |
| --- | --- | --- | --- | --- |
| **RSV** | 1 | Exp Respiratory Syncytial Virus, Human/ | 3028 | 3476 |
|  | 2 | (respiratory syncytial virus or RSV).ti,ab,kf. | 19038 | 20065 |
|  | 3 | 1 or 2 | 19155 | 20182 |
| **UK (NICE filter)** | 4 | exp United Kingdom/ | 379321 | 385851 |
|  | 5 | (national health service$ or nhs$).ti,ab,in. | 229083 | 250008 |
|  | 6 | (english not ((published or publication$ or translat$ or written or language$ or speak$ or literature or citation$) adj5 english)).ti,ab. | 41976 | 45573 |
|  | 7 | (gb or "g.b." or britain$ or (british$ not "british columbia") or uk or "u.k." or united kingdom$ or (england$ not "new england") or northern ireland$ or northern irish$ or scotland$ or scottish$ or ((wales or "south wales") not "new south wales") or welsh$).ti,ab,jw,in. | 2230097 | 2336480 |
|  | 8 | (Aberdeen or Armagh or bangor or bath or Belfast or Birmingham or Bradford or brighton or Bristol or Cambridge or Canterbury or Cardiff or Carlisle or Chelmsford or chester or Chichester or Coventry or derby or derry or Dundee or durham or Edinburgh or ely or Exeter or Glasgow or Gloucester or Hereford or inverness or Kingston or Lancaster or leeds or Leicester or Lichfield or Lincoln or Lisburn or Liverpool or London or Manchester or Newcastle or Newport or Newry or Norwich or Nottingham or oxford or perth or Peterborough or Plymouth or Portsmouth or preston or ripon or St Albans or St Asaph or St David's or Salford or Salisbury or Sheffield or Southampton or Stirling or Stoke or Sunderland or Swansea or Truro or Wakefield or Wells or Westminster or Winchester or Wolverhampton or Worcester or York).ti,ab,in. | 2753712 | 2919010 |
|  | 9 | or/4-8 | 3720827 | 3908454 |
|  | 10 | (exp antarctic regions/ or exp arctic regions/ or exp Americas/ or exp oceania/ or exp africa/ or exp asia/) not (exp united kingdom/ or europe/) | 3088895 | 3227601 |
|  | 11 | 9 not 10 | 3451563 | 3615606 |
| **Combined** | 12 | 3 and 11 | 2427 | 2540 |
| **Exclusion terms** | 13 | exp animals/ not exp humans/ | 4895072 | 5035000 |
|  | 14 | (comment or editorial or case reports or historical article).pt. | 3876471 | 4011589 |
|  | 15 | comment/ or editorial/ or case reports/ or historical article/ | 3876471 | 4011589 |
|  | 16 | (case stud$ or case report$).ti. | 329028 | 353215 |
|  | 17 | or/13-16 | 8761258 | 9036785 |
|  | 18 | 12 not 17 | 1904 | 1998 |
| **Date limit** | 19 | limit 18 to yr=2011-current | **1,038** | **1,132** |

**Footnotes**: Records deduplicated against results from other electronic databases and records from the update searches additionally deduplicated against the original SLR database hits. Only novel records were carried forward for title/abstract screening.

**Database:** Ovid MEDLINE(R) and Epub Ahead of Print, In-Process & Other Non-Indexed Citations and Daily 1946 to October 6, 2021 (original SLR) and 1964 to August 5, 2022 (SLR update).

Table S2. Search terms for use in Embase (searched via the Ovid SP platform)

| **Term Group** | **#** | **Search Terms** | **Original SLR Results (07/10/2021)** | **SLR Update Results (08/08/2022)** |
| --- | --- | --- | --- | --- |
| **RSV** | 1 | Exp Human respiratory syncytial virus/ | 6086 | 7153 |
|  | 2 | (respiratory syncytial virus or RSV).ti,ab,kf. | 24164 | 25455 |
|  | 3 | 1 or 2 | 25607 | 27353 |
| **UK (NICE filter)** | 4 | exp Great Britain/ or exp United Kingdom/ | 435256 | 447194 |
|  | 5 | (national health service$ or nhs$).ti,ab,in. | 337707 | 366319 |
|  | 6 | (english not ((published or publication$ or translat$ or written or language$ or speak$ or literature or citation$) adj5 english)).ti,ab. | 49636 | 54148 |
|  | 7 | (gb or "g.b." or britain$ or (british$ not "british columbia") or uk or "u.k." or united kingdom$ or (england$ not "new england") or northern ireland$ or northern irish$ or scotland$ or scottish$ or ((wales or "south wales") not "new south wales") or welsh$).ti,ab,in,ad. | 3063139 | 3191440 |
|  | 8 | (Aberdeen or Armagh or bangor or bath or Belfast or Birmingham or Bradford or brighton or Bristol or Cambridge or Canterbury or Cardiff or Carlisle or Chelmsford or chester or Chichester or Coventry or derby or derry or Dundee or durham or Edinburgh or ely or Exeter or Glasgow or Gloucester or Hereford or inverness or Kingston or Lancaster or leeds or Leicester or Lichfield or Lincoln or Lisburn or Liverpool or London or Manchester or Newcastle or Newport or Newry or Norwich or Nottingham or oxford or perth or Peterborough or Plymouth or Portsmouth or preston or ripon or St Albans or St Asaph or St David's or Salford or Salisbury or Sheffield or Southampton or Stirling or Stoke or Sunderland or Swansea or Truro or Wakefield or Wells or Westminster or Winchester or Wolverhampton or Worcester or York).ti,ab,in,ad. | 4398465 | 4591594 |
|  | 9 | or/4-8 | 5206616 | 5426407 |
|  | 10 | (exp antarctica/ or exp arctic/ or exp "arctic and Antarctic"/ or exp oceanic regions/ or exp western hemisphere/ or exp africa/ or exp asia/) not (exp united kingdom/ or exp great Britain/ or europe/) | 3167136 | 3315476 |
|  | 11 | 9 not 10 | 4858480 | 5056082 |
| **Combined** | 12 | 3 and 11 | 3845 | 4084 |
| **Exclusion terms** | 13 | ("conference abstract" or "conference review").pt. | 4223569 | 4498326 |
|  | 14 | limit 13 to yr="1974-2018" | 3399650 | 3404607 |
|  | 15 | exp animals/ not exp humans/ | 4845020 | 4980741 |
|  | 16 | (editorial).pt. | 7034842 | 733138 |
|  | 17 | comment/ or editorial/ or case reports/ or historical article/ | 681871 | 704237 |
|  | 18 | (case stud$ or case report$).ti. | 402461 | 429377 |
|  | 19 | or/14-18 | 9017488 | 9212289 |
|  | 20 | 12 not 19 | 2737 | 2958 |
| **Date limit** | 21 | limit 20 to yr=2011-current | **1,432** | **1,657** |

**Footnotes**: Records deduplicated against results from other electronic databases and records from the update searches additionally deduplicated against the original SLR database hits. Only novel records were carried forward for title/abstract screening.

**Database:** Embase 1974 to 2021 October 6 (original SLR), and 1974 to 2022 August 5 (SLR update)

Table S3. Search terms for use in the CDSR and CENTRAL databases (searched via the Wiley platform)

| **Term Group** | **#** | **Search Terms** | **Original SLR Results (07/10/2021)** | **SLR Update Results (08/08/2022)** |
| --- | --- | --- | --- | --- |
| **RSV** | 1 | [mh "Respiratory Syncytial Virus, Human"] | 60 | 77 |
|  | 2 | (respiratory syncytial virus or RSV):ti,ab,kw | 1238 | 1312 |
|  | 3 | #1 or #2 | 1238 | 1312 |
| **UK (NICE filter)** | 4 | [mh "United Kingdom"] | 6674 | 6845 |
|  | 5 | ((national health NEXT service*) or nhs*):ti,ab | 5963 | 6321 |
|  | 6 | (english not ((published or publication* or translat* or written or language* or speak* or literature or citation*) NEAR/5 english)):ti,ab | 3364 | 3693 |
|  | 7 | (gb or "g.b. " or Britain* or (British* not "british columbia") or uk or "u.k." or (united NEXT kingdom*) or (England* not "new england") or (northern NEXT Ireland*) or (northern NEXT irish*) or Scotland* or Scottish* or ((wales or "south wales") not "new south wales") or welsh*):ti,ab | 31828 | 32837 |
|  | 8 | (Aberdeen or Armagh or bangor or bath or Belfast or Birmingham or Bradford or brighton or Bristol or Cambridge or Canterbury or Cardiff or Carlisle or Chelmsford or chester or Chichester or Coventry or derby or derry or Dundee or durham or Edinburgh or ely or Exeter or Glasgow or Gloucester or Hereford or inverness or Kingston or Lancaster or leeds or Leicester or Lichfield or Lincoln or Lisburn or Liverpool or London or Manchester or Newcastle or Newport or Newry or Norwich or Nottingham or oxford or perth or Peterborough or Plymouth or Portsmouth or preston or ripon or St Albans or St Asaph or St David's or Salford or Salisbury or Sheffield or Southampton or Stirling or Stoke or Sunderland or Swansea or Truro or Wakefield or Wells or Westminster or Winchester or Wolverhampton or Worcester or York):ti,ab | 33703 | 35368 |
|  | 9 | {or #4-#8} | 66232 | 69146 |
|  | 10 | ([mh "antarctic regions"] or [mh "arctic regions"] or [mh "Americas"] or [mh "oceania"] or [mh "Africa"] or [mh "asia"]) not ([mh "united kingdom"] or [mh ^"Europe"]) | 59056 | 62590 |
|  | 11 | #9 not #10 | 64406 | 67199 |
| **Combined and total** | 12 | #3 and #11 | 32 | 33 |
|  | 13 | #12 with Cochrane Library publication date between Jan 2011 to Dec 2021 | **23** | **20** |

**Footnotes**: Records deduplicated against results from other electronic databases and records from the update searches additionally deduplicated against the original SLR database hits. Only novel records were carried forward for title/abstract screening.

**Database:** Cochrane Database of Systematic Reviews, Issue 10 of 12, 2021 (original SLR) and Issue 8 of 12, 2022 (SLR update); Cochrane Central Register of Controlled Trials, Issue 10 of 12, 2021 (original SLR) and Issue 7 of 12, 2022 (SLR update).

Table S4. Search terms for use in DARE and the NHS-EED (searched via the York CRD platform)

| **Term Group** | **#** | **Search Terms** | **Original SLR Results (07/10/2021)** |
| --- | --- | --- | --- |
| **RSV** | 1 | MeSH DESCRIPTOR Respiratory Syncytial Virus, Human EXPLODE ALL TREES | 10 |
|  | 2 | (respiratory syncytial virus or RSV) | 101 |
|  | 3 | #1 or #2 | 101 |
| **UK (NICE filter)** | 4 | MeSH DESCRIPTOR United Kingdom EXPLODE ALL TREES | 498 |
|  | 5 | (national health service* or nhs*) | 12466 |
|  | 6 | (english not (((published or publication* or translat* or written or language* or speak* or literature or citation*) adj5 english) or (english adj5 (published or publication* or translat* or written or language* or speak* or literature or citation*)))) | 30031 |
|  | 7 | (gb or "g b " or Britain* or (British* not "british columbia") or uk or "u k " or united kingdom* or (England* not "new england") or northern Ireland* or northern irish* or Scotland* or Scottish* or ((wales or "south wales") not "new south wales") or welsh*) | 20694 |
|  | 8 | (Aberdeen or Armagh or bangor or bath or Belfast or Birmingham or Bradford or brighton or Bristol or Cambridge or Canterbury or Cardiff or Carlisle or Chelmsford or chester or Chichester or Coventry or derby or derry or Dundee or durham or Edinburgh or ely or Exeter or Glasgow or Gloucester or Hereford or inverness or Kingston or Lancaster or leeds or Leicester or Lichfield or Lincoln or Lisburn or Liverpool or London or Manchester or Newcastle or Newport or Newry or Norwich or Nottingham or oxford or perth or Peterborough or Plymouth or Portsmouth or preston or ripon or St Albans or St Asaph or St David's or Salford or Salisbury or Sheffield or Southampton or Stirling or Stoke or Sunderland or Swansea or Truro or Wakefield or Wells or Westminster or Winchester or Wolverhampton or Worcester or York) | 30031 |
|  | 9 | #4 OR #5 OR #6 OR #7 OR #8 | 42721 |
|  | 10 | MeSH DESCRIPTOR antarctic regions EXPLODE ALL TREES | 0 |
|  | 11 | MeSH DESCRIPTOR arctic regions EXPLODE ALL TREES | 0 |
|  | 12 | MeSH DESCRIPTOR americas EXPLODE ALL TREES | 4408 |
|  | 13 | MeSH DESCRIPTOR oceania EXPLODE ALL TREES | 489 |
|  | 14 | MeSH DESCRIPTOR africa EXPLODE ALL TREES | 559 |
|  | 15 | MeSH DESCRIPTOR asia EXPLODE ALL TREES | 1436 |
|  | 16 | #10 or #11 OR #12 OR #13 OR #14 OR #15 | 6721 |
|  | 17 | MeSH DESCRIPTOR Europe | 253 |
|  | 18 | #4 or #17 | 750 |
|  | 19 | #16 not #18 | 6621 |
|  | 20 | #9 not #19 | 36987 |
| **Combined and total** | 21 | #3 and #20 | 55 |
|  | 22 | #21 FROM 2011 TO 2021, in NHSEED, DARE | **7** |

**Footnotes:** DARE and NHS EED were only searched in the original SLR. These databases were not re-searched in the SLR update as the databases were last updated in April 2015, and are no longer maintained, so would not contain any novel records to review.

**Database(s):** NHS Economic Evaluation Database: Issue 2 of 4, April 2015; Database of Abstracts of Reviews of Effect: Issue 2 of 4, April 2015.

Table S5. Search terms for the International HTA Database (searched via the INAHTA platform)

| **Term Group** | **#** | **Search Terms** | **Original SLR Results (07/10/2021)** | **SLR Update Results (09/08/2022)** |
| --- | --- | --- | --- | --- |
| **RSV** | 1 | “Respiratory Syncytial Virus, Human”[mhe] | 5 | 5 |
|  | 2 | (respiratory syncytial virus or RSV) | 19 | 20 |
|  | 3 | #1 or #2 | 19 | 20 |
| **UK (NICE filter)** | 4 | “United Kingdom”[mhe] | 3 | 70 |
|  | 5 | (national health service* or nhs*) | 1706 | 1800 |
|  | 6 | (english not ((published or publication* or translat* or written or language* or speak* or literature or citation*) and english)) | 13627 | 14032 |
|  | 7 | (gb or "g.b." or Britain* or (British* not "british columbia") or uk or "u.k." or united kingdom* or (England* not "new england") or northern Ireland* or northern irish* or Scotland* or Scottish* or ((wales or "south wales") not "new south wales") or welsh*) | 9330 | 9430 |
|  | 8 | (Aberdeen or Armagh or bangor or bath or Belfast or Birmingham or Bradford or brighton or Bristol or Cambridge or Canterbury or Cardiff or Carlisle or Chelmsford or chester or Chichester or Coventry or derby or derry or Dundee or durham or Edinburgh or ely or Exeter or Glasgow or Gloucester or Hereford or inverness or Kingston or Lancaster or leeds or Leicester or Lichfield or Lincoln or Lisburn or Liverpool or London or Manchester or Newcastle or Newport or Newry or Norwich or Nottingham or oxford or perth or Peterborough or Plymouth or Portsmouth or preston or ripon or St Albans or St Asaph or St David's or Salford or Salisbury or Sheffield or Southampton or Stirling or Stoke or Sunderland or Swansea or Truro or Wakefield or Wells or Westminster or Winchester or Wolverhampton or Worcester or York) | 375 | 392 |
|  | 9 | #4 OR #5 OR #6 OR #7 OR #8 | 14251 | 14731 |
|  | 10 | (“antarctic regions”[mhe]) OR (“arctic regions”[mhe]) OR (“Americas”[mhe]) OR (“oceania”[mhe] ) OR (“Africa”[mhe]) OR (“asia”[mhe] ) | 233 | 227 |
|  | 11 | (“Europe”[mh]) | 11 | 13 |
|  | 12 | #4 or #11 | 14 | 82 |
|  | 13 | #10 not #12 | 229 | 182 |
|  | 14 | #9 not #13 | 9706 | 6100 |
| **Combined** | 15 | #3 and #14 FROM 2011 TO 2021 | **7** | **3** |

**Footnotes**: Records deduplicated against results from other electronic databases and records from the update searches additionally deduplicated against the original SLR database hits. Only novel records were carried forward for title/abstract screening.

**Database:** International HTA Database searched 7 October 2021 (original SLR) and 9 August 2022 (SLR update).

Grey Literature Searching

Conference Proceedings

Conference proceedings from the following conferences were manually hand searched to identify any relevant abstracts for the SLR:

- European Society of Clinical Microbiology and Infectious Diseases (ESCMID) Annual Meeting
  - 29th European Congress of Clinical Microbiology and Infectious Disease 2019, Amsterdam, available at: https://2019.eccmid.org/
  - 30th European Congress of Clinical Microbiology and Infectious Disease 2020, Paris, available at: https://2020.eccmid.org/
  - 31st European Congress of Clinical Microbiology and Infectious Disease 2022, Lisbon, available at: https://www.eccmid.org/
- European Respiratory Society (ERS) Annual Meeting
  - ERS International Congress 2019, Madrid, available at: https://erj.ersjournals.com/content/54/suppl_63
  - ERS International Congress 2020, Virtual, available at: https://erj.ersjournals.com/content/56/suppl_64
  - ERS International Congress 2021, Virtual, available at: https://erj.ersjournals.com/content/58/suppl_65
- IDWeek Annual Meeting
  - IDWeek 2019, Washington, available at: https://academic.oup.com/ofid/issue/6/Supplement_2
  - IDWeek 2020, Virtual, available at: https://academic.oup.com/ofid/issue/7/Supplement_1
  - IDWeek 2021, Virtual, available at: https://academic.oup.com/ofid/issue/8/Supplement_1
- The International Society for Pharmacoeconomics and Outcomes Research (ISPOR) Annual International and European Meetings, all available at: https://www.ispor.org/heor-resources/presentations-database/search
  - ISPOR 2019, New Orleans
  - ISPOR Europe 2019, Copenhagen
  - ISPOR 2020, Orlando
  - ISPOR Europe 2020, Milan
  - ISPOR 2021, Montreal
  - ISPOR Europe 2021, Copenhagen
  - ISPOR 2022, Washington DC

The search strategies used to search the conferences are presented in Table S6. The conference ECCMID 2021 could not be searched as the abstracts could not be accessed.

Table S6. Search strategies for hand-searching of relevant congresses in the original SLR and SLR update

| **Congress** | **Year** | **Search Strategy** | **Number of Hits** | **Number of Relevant Hits** |
| --- | --- | --- | --- | --- |
| **European Society of Clinical Microbiology and Infectious Diseases (ESCMID) Annual Meeting*** | 2019 | Search “RSV” and screen the results against eligibility criteria. | 12 | 0 |
|  |  | Search “respiratory syncytial virus” and screen the results against eligibility criteria. | 67 | 0 |
|  | 2020 | Search “RSV” in the search bar; date limit the library to time period: 01/01/2019 - 12/31/2019 and screen the results against eligibility criteria. | 72 | 0 |
|  |  | Search “respiratory syncytial virus” in the search bar; date limit the library to time period: 01/01/2019 - 12/31/2019 and screen the results against eligibility criteria. |  | 0 |
|  | 2022 | Title sift of abstracts containing “RSV” or “respiratory syncytial virus”. In addition a title sift of all abstracts under the following headings was performed:   1. OF222 Viral respiratory infections: influenza and RSV 2. PS003 1c. Influenza and respiratory viruses (incl diagnostics & epidemiology, antiviral drugs, vaccines, treatment & susceptibility/resistance)   Any relevant abstracts at title stage had the full abstract reviewed. | 42 | 0 |
| **European Respiratory Society (ERS) Annual Meeting** | 2019 | Search “RSV” in the 'search within this issue' search bar and screen the results against eligibility criteria. | 26 | 0 |
|  |  | Search “respiratory syncytial virus” in the 'search within this issue' search bar and screen the results against eligibility criteria. | 21 | 0 |
|  | 2020 | Search “RSV” in the 'search within this issue' search bar and screen the results against eligibility criteria. | 9 | 0 |
|  |  | Search “respiratory syncytial virus” in the 'search within this issue' search bar and screen the results against eligibility criteria. | 7 | 0 |
|  | 2021 | Search “RSV” in the 'search within this issue' search bar and screen the results against eligibility criteria. | 15 | 0 |
|  |  | Search “respiratory syncytial virus” in the 'search within this issue' search bar and screen the results against eligibility criteria. | 10 | 0 |
| **IDWeek Annual Meeting** | 2019 | Use the search bar 'keyword search' to search “RSV”; review all records underneath the tab 'presentations', 'posters' and 'poster presenters' | 16 | 0 |
|  |  | Use the search bar 'keyword search' to search “respiratory syncytial virus”; review all records underneath the tab 'presentations', 'posters' and 'poster presenters' | 4 | 0 |
|  | 2020 | Use the search bar 'keyword search' to search “RSV”; review all records underneath the tab 'presentations', 'posters' and 'poster presenters' | 23 | 0 |
|  |  | Use the search bar 'keyword search' to search “respiratory syncytial virus”; review all records underneath the tab 'presentations', 'posters' and 'poster presenters' | 21 | 0 |
|  | 2021 | Use the search bar 'keyword search' to search “RSV”; review all records underneath the tab 'presentations', 'posters' and 'poster presenters' | 10 | 0 |
|  |  | Use the search bar 'keyword search' to search “respiratory syncytial virus”; review all records underneath the tab 'presentations', 'posters' and 'poster presenters' | 19 | 0 |
| **The International Society for Pharmacoeconomics and Outcomes Research (ISPOR) Europe Annual Meeting** | 2019 | Select conference as 2021-11 ISPOR Europe 2021 conference and enter search strategy “RSV or respiratory syncytial virus” into the 'keywords' box. | 4 | 0 |
|  | 2020 | Select conference as 2021-11 ISPOR Europe 2021 conference and enter search strategy “RSV or respiratory syncytial virus” into the 'keywords' box. | 5 | 0 |
|  | 2021 | Select conference as 2021-11 ISPOR Europe 2021 conference and enter search strategy “RSV or respiratory syncytial virus” into the 'keywords' box. | 35 | 0 |
| **The International Society for Pharmacoeconomics and Outcomes Research (ISPOR) International Annual Meeting** | 2019 | Select conference as 2021-05 ISPOR 2021 conference and enter search strategy “RSV or respiratory syncytial virus” into the 'keywords' box | 0 | 0 |
|  | 2020 | Select conference as 2021-05 ISPOR 2021 conference and enter search strategy “RSV or respiratory syncytial virus” into the 'keywords' box | 2 | 0 |
|  | 2021 | Select conference as 2021-05 ISPOR 2021 conference and enter search strategy “RSV or respiratory syncytial virus” into the 'keywords' box | 13 | 0 |
|  | 2022 | Select conference as 2022-05 ISPOR 2022 conference and enter search strategy “RSV or respiratory syncytial virus” into the 'keywords' box | 6 | 0 |

**Footnote:** *ESCMID 2021 was unable to be searched as the abstracts were unavailable.

**Abbreviations:** ERS: European Respiratory Society; ESCMID: European Society of Clinical Microbiology and Infectious Diseases; ISPOR: International Society for Pharmacoeconomics and Outcomes Research; RSV: respiratory syncytial virus.

Economic Website and HTA Searches

To supplement the search of electronic databases and hand searching sources detailed above, the following UK HTA body websites were searched, in the original SLR, to identify HTAs published since 2011, from which relevant data could be extracted. For the SLR update the same searches were conducted to identify any records published or updated since the original SLR was conducted.

- National Institute for Health and Care Excellence (NICE), available at: https://www.nice.org.uk/
- Scottish Medicines Consortium (SMC), available at: https://www.scottishmedicines.org.uk/
- All Wales Medicine Strategy Group (AWMSG), available at: https://awmsg.nhs.wales/

For the original SLR, in line with current best practice guidelines for identifying economic inputs relevant to cost-effectiveness modelling, the Cost-Effectiveness Analysis (CEA) Registry, managed by Tufts Medical Center, was also searched (available at: http://healtheconomics.tuftsmedicalcenter.org/cear2n/search/search.aspx). Since the completion of the original SLR, the CEA registry had changed platforms, and so the new platform was searched for articles published since 2011 in the SLR update (available at: https://cear.tuftsmedicalcenter.org/). The search strategies for HTA websites are presented in Table S7.

The search strategies used to search the economic websites are presented in Table S8 and Table S9, for the original SLR and SLR update, respectively.

Table S7. Search strategy for HTA website searches

| HTA Website | Search Strategy | Number of Hits in Original SLR (28/10/21) | Number of Hits in SLR Update (11/08/22) | Number of Relevant Hits |
| --- | --- | --- | --- | --- |
| All Wales Medicines Strategy Group (AWMSG)  http://www.awmsg.org/ | Search “RSV” in search bar. Screen the results against eligibility criteria. For each relevant result, review the “AWMSG Secretariat Appraisal Report (ASAR)” document for relevance. | 1 | 1 | 0 |
|  | Search “respiratory syncytial virus” in search bar. Screen the results against eligibility criteria. For each relevant result, review the “AWMSG Secretariat Appraisal Report (ASAR)” document for relevance. | 141 | 164 | 0 |
| Scottish Medicines Consortium (SMC)  https://www.scottishmedicines.org.uk/Home | Search “RSV” in search bar. Screen the results against eligibility criteria. | 0 | 0 | 0 |
|  | Search “respiratory syncytial virus” in search bar. Screen the results against eligibility criteria. | 0 | 0 | 0 |
| National Institute for Health and Care Excellence (NICE)  https://www.nice.org.uk/ | Search “RSV” in search bar filtered for “Guidance” and “NICE advice”. Screen “Final Appraisal Document” and “Committee papers” for each record for relevance. | 2 | 2 | 0 |
|  | Search “respiratory syncytial virus” in search bar filtered for “Guidance” and “NICE advice”. Screen “Final Appraisal Document” and “Committee papers” for each record for relevance. | 2 | 2 | 0 |

**Abbreviations**: AWMSG: All Wales Medicines Strategy Group; HTA: Health Technology Assessment; NICE: National Institute for Health and Care Excellence; RSV; respiratory syncytial virus; SMC: Scottish Medicines Consortium.

Table S8. Search strategy for economic website searches in original SLR

| Economic Website | Search Strategy | Number of Hits in original SLR (28/10/21) | Number of Relevant Hits |
| --- | --- | --- | --- |
| The Cost-Effectiveness Analysis (CEA) Registry  http://healtheconomics.tuftsmedicalcenter.org/cear2n/search/search.aspx | Search “RSV” in search bar. Select 'methods' and review all articles published since 2011 for relevance. | 14 | 0 |
|  | Search “respiratory syncytial virus” in search bar. Select 'methods' and review all articles published since 2011 for relevance. | 14 | 0 |
|  | Search “RSV” in search bar. Select 'ratios' and review all articles published since 2011 for relevance. | 39 | 0 |
|  | Search “respiratory syncytial virus” in search bar. Select 'ratios' and review all articles published since 2011 for relevance. | 38 | 0 |

**Abbreviations**: CEA: Cost-Effectiveness Analysis; RSV; respiratory syncytial virus.

Table S9. Search strategy for economic website searches in SLR update

| HTA Website | Search Strategy | Number of Hits in SLR update (11/08/22) | Number of Relevant Hits |
| --- | --- | --- | --- |
| The Cost-Effectiveness Analysis (CEA) Registry  https://cear.tuftsmedicalcenter.org/ | Search “RSV” in search bar. Select 'methods' and review all articles published since 2011 for relevance. | 17 | 0 |
|  | Search “respiratory syncytial virus” in search bar. Select 'methods' and review all articles published since 2011 for relevance. | 17 | 0 |
|  | Search “RSV” in search bar. Select 'ratios' and review all articles published since 2011 for relevance. | 6 | 0 |
|  | Search “respiratory syncytial virus” in search bar. Select 'ratios' and review all articles published since 2011 for relevance. | 11 | 0 |

**Abbreviations:** CEA: Cost-Effectiveness Analysis; RSV; respiratory syncytial virus.

Bibliography Searches

The bibliographies of all relevant SLRs, NMAs, economic evaluations, and HTAs identified during the original SLR and SLR update were hand-searched in order to identify any additional, relevant studies for inclusion.

Appendix S2. Full List of Studies Included in the SLR

Table S10. List of studies included in the SLR

| **Study** | **Title** | **Journal** |
| --- | --- | --- |
| Balassa et al. 2019^1^ | Treatment stratification of respiratory syncytial virus infection in allogeneic stem cell transplantation | Journal of Infection |
| Berry et al. 2020^2^ | Point of care testing of Influenza A/B and RSV in an adult respiratory assessment unit is associated with improvement in isolation practices and reduction in hospital length of stay | Journal of Medical Microbiology |
| Blackburn et al. 2018^3^ | Laboratory-confirmed respiratory infections as predictors of hospital admission for myocardial infarction and stroke: Time-series analysis of English data for 2004-2015 | Clinical Infectious Diseases |
| Chiavenna et al. 2019^4^ | Estimating age-stratified influenza-associated invasive pneumococcal disease in england: A time-series model based on population surveillance data | PLoS Medicine |
| Dadhwal et al. 2022^5^ | Severe COVID-19 pneumonia in an intensive care setting and comparisons with historic severe viral pneumonia due to other viruses | Clinical Respiratory Journal |
| Fleming et al. 2015^6^ | Modelling estimates of the burden of Respiratory Syncytial virus infection in adults and the elderly in the United Kingdom | BMC Infectious Diseases |
| Flight et al. 2014^7^ | Incidence and clinical impact of respiratory viruses in adults with cystic fibrosis | Thorax |
| Gadsby et al. 2016^8^ | Comprehensive molecular testing for respiratory pathogens in community-acquired pneumonia | Clinical Infectious Diseases |
| Gorcea et al. 2017^9^ | Effective use of oral ribavirin for respiratory syncytial viral infections in allogeneic haematopoietic stem cell transplant recipients | Journal of Hospital Infection |
| Green et al. 2013^10^ | Critical care surveillance: insights into the impact of the 2010/11 influenza season relative to the 2009/10 pandemic season in England | Euro Surveillance: Bulletin Europeen sur les Maladies Transmissibles = European Communicable Disease Bulletin |
| Hodgson et al. 2020^11^ | Evaluating the next generation of RSV intervention strategies: a mathematical modelling study and cost-effectiveness analysis | BMC Medicine |
| Hughes et al. 2016^12^ | Emergency department syndromic surveillance providing early warning of seasonal respiratory activity in England | Epidemiology and Infection |
| Inkster et al. 2017^13^ | Consecutive yearly outbreaks of respiratory syncytial virus in a haemato-oncology ward and efficacy of infection control measures | Journal of Hospital Infection |
| Johannesen et al. 2022^14^ | Age-Specific Estimates of Respiratory Syncytial Virus-Associated Hospitalizations in 6 European Countries: A Time Series Analysis | Journal of Infectious Diseases |
| Korsten et al. 2021^15^ | Burden of respiratory syncytial virus infection in community-dwelling older adults in Europe (RESCEU): an international prospective cohort study | European Respiratory Journal |
| Morbey et al. 2017^16^ | The burden of seasonal respiratory infections on a national telehealth service in England | Epidemiology and Infection |
| Morbey et al. 2018^17^ | Estimating the burden on general practitioner services in England from increases in respiratory disease associated with seasonal respiratory pathogen activity | Epidemiology and Infection |
| Morris et al. 2021^18^ | Xpert Xpress Flu/RSV: Validation and impact evaluation at a large UK hospital trust | Journal of Medical Virology |
| Partridge et al. 2022^19^ | Universal use of surgical masks is tolerated and prevents respiratory viral infection in stem cell transplant recipients | Journal of Hospital Infection |
| Ponsford et al. 2021^20^ | Increased Respiratory Viral Detection and Symptom Burden Among Patients with Primary Antibody Deficiency: Results from the BIPAD Study | Journal of Allergy and Clinical Immunology: In Practice |
| Poole et al. 2020^21^ | SARS-CoV-2 has displaced other seasonal respiratory viruses: Results from a prospective cohort study | Journal of Infection |
| Russell et al. 2018^22^ | Challenges in the diagnosis of leptospirosis outwith endemic settings: A scottish single centre experience | Journal of the Royal College of Physicians of Edinburgh |
| Sharp et al. 2022^23^ | Estimating the burden of adult hospital admissions due to RSV and other respiratory pathogens in England | Influenza and other Respiratory Viruses |
| Swets et al. 2022^24^ | SARS-CoV-2 co-infection with influenza viruses, respiratory syncytial virus, or adenoviruses | Lancet |
| Tanner et al. 2012^25^ | Respiratory viral infections during the 2009-2010 winter season in Central England, UK: Incidence and patterns of multiple virus co-infections | European Journal of Clinical Microbiology and Infectious Diseases |
| Weir-Mccall et al. 2022^26^ | Vascular Thrombosis in Severe COVID-19 Requiring Extracorporeal Membrane Oxygenation: A Multicenter Study | Critical Care Medicine |
| Youngs et al. 2019^27^ | Implementation of the cobas Liat influenza point-of-care test into an emergency department during a high-incidence season: a retrospective evaluation following real-world implementation | Journal of Hospital Infection |
| Zhao et al. 2014^28^ | A new laboratory-based surveillance system (Respiratory DataMart System) for influenza and other respiratory viruses in England: results and experience from 2009 to 2012 | Euro Surveillance: Bulletin Europeen sur les Maladies Transmissibles = European Communicable Disease Bulletin |

Appendix S3. Full Lists of Studies Excluded at Full-Text Review Stage

The full list of studies excluded at the full-text review stage is presented below in Table S11 and Table S12 for the original SLR and the SLR update respectively.

Table S11. List of studies excluded at the full-text stage review in the original SLR

| **Study** | **Title** | **Journal** | **Reason for Exclusion** |
| --- | --- | --- | --- |
| Agoti et al. 2015^29^ | Successive respiratory syncytial virus epidemics in local populations arise from multiple variant introductions, providing insights into virus persistence | Journal of Virology | a |
| Arnold et al. 2019^30^ | The role of viruses in the development of pleural infection | European Respiratory Journal. Conference: 29th International Congress of the European Respiratory Society, ERS. Madrid Spain | b |
| Atabani et al. 2016^31^ | Active screening and surveillance in the United Kingdom for Middle East respiratory syndrome coronavirus in returning travellers and pilgrims from the Middle East: A prospective descriptive study for the period 2013-2015 | International Journal of Infectious Diseases | c |
| Bailey et al. 2019^32^ | A mini-review of adverse lung transplant outcomes associated with respiratory viruses | Frontiers in Immunology | d |
| Bhaskaran et al. 2011^33^ | The effects of hourly differences in air pollution on the risk of myocardial infarction: Case crossover analysis of the MINAP database | BMJ (Online) | c |
| Bouzid et al. 2021^34^ | Systematic review on the association between respiratory virus real-time PCR cycle threshold values and clinical presentation or outcomes | Journal of Antimicrobial Chemotherapy | d |
| Brittain-Long et al. 2012^35^ | Seasonal variations of 15 respiratory agents illustrated by the application of a multiplex polymerase chain reaction assay | Scandinavian Journal of Infectious Diseases | b |
| Broberg et al. 2018^36^ | Seasonality and geographical spread of respiratory syncytial virus epidemics in 15 european countries, 2010 to 2016 | Eurosurveillance | c |
| Broor et al. 2020^37^ | Leveraging the Global Influenza Surveillance and Response System for global respiratory syncytial virus surveillance – Opportunities and challenges | Influenza and Other Respiratory Viruses | c |
| Brunwasser et al. 2020^38^ | Assessing the strength of evidence for a causal effect of respiratory syncytial virus lower respiratory tract infections on subsequent wheezing illness: A systematic review and meta-analysis | The Lancet Respiratory Medicine | a |
| Bruyndonckx et al. 2020^39^ | Respiratory syncytial virus and influenza virus infection in adult primary care patients: Association of age with prevalence, diagnostic features and illness course | International Journal of Infectious Diseases | c |
| Chadha et al. 2020^40^ | Human respiratory syncytial virus and influenza seasonality patterns – Early findings from the WHO global respiratory syncytial virus surveillance | Influenza and Other Respiratory Viruses | a |
| Coughtrie et al. 2018^41^ | Ecology and diversity in upper respiratory tract microbial population structures from a cross-sectional community swabbing study | Journal of Medical Microbiology | c |
| D'Anna et al. 2020^42^ | Evaluation of innate immune mediators related to respiratory viruses in the lung of stable COPD patients | Journal of Clinical Medicine | b |
| Dolby et al. 2021^43^ | Histological evidence of pulmonary microthrombosis and vasculitis in life-threatening respiratory virus diseases | Open Forum Infectious Diseases | d |
| Eyre et al. 2021^44^ | Reduction in incidence of non-COVID-19 respiratory virus infection amongst haematology inpatients following UK social distancing measures | British Journal of Haematology | c |
| Falsey et al. 2014^45^ | Respiratory syncytial virus and other respiratory viral infections in older adults with moderate to severe influenza-like illness | Journal of Infectious Diseases | d |
| Falsey et al. 2019^46^ | Respiratory syncytial virus-associated illness in adults with advanced chronic obstructive pulmonary disease and/or congestive heart failure | Journal of Medical Virology | b |
| Falsey et al. 2022^47^ | Comparative assessment of reported symptoms of influenza, respiratory syncytial virus, and human metapneumovirus infection during hospitalization and post-discharge assessed by Respiratory Intensity and Impact Questionnaire | Influenza and Other Respiratory Viruses | b |
| Felt et al. 2021^48^ | Detection of respiratory syncytial virus defective genomes in nasal secretions is associated with distinct clinical outcomes | Nature Microbiology | d |
| Feng et al. 2020^49^ | Effects of cinnamaldehyde on anti-respiratory syncytial virus: A protocol of systematic review and meta-analysis | Medicine (United States) | d |
| French et al. 2016^50^ | Risk of nosocomial respiratory syncytial virus infection and effectiveness of control measures to prevent transmission events: A systematic review | Influenza and Other Respiratory Viruses | d |
| Gadsby et al. 2019^51^ | Comparison of Unyvero P55 Pneumonia Cartridge, in-house PCR and culture for the identification of respiratory pathogens and antibiotic resistance in bronchoalveolar lavage fluids in the critical care setting | European Journal of Clinical Microbiology and Infectious Diseases | a |
| Gaunt et al. 2011^52^ | Disease burden of the most commonly detected respiratory viruses in hospitalized patients calculated using the disability adjusted life year (DALY) model | Journal of Clinical Virology | c |
| Goka et al. 2013^53^ | Influenza A viruses dual and multiple infections with other respiratory viruses and risk of hospitalisation and mortality | Influenza and Other Respiratory Viruses | c |
| Goka et al. 2015^54^ | Single, dual and multiple respiratory virus infections and risk of hospitalization and mortality | Epidemiology and Infection | c |
| Goldstein et al. 2020^55^ | Impact of implementing respiratory point-of-care testing in a regional haemato-oncology unit | Journal of Hospital Infection | a |
| Goldstein et al. 2019^56^ | In-house validation of the cobas Liat influenza A/B and RSV assay for use with gargles, sputa and endotracheal secretions | Journal of Hospital Infection | d |
| Green et al. 2013^10^ | Mortality attributable to influenza in England and Wales prior to, during and after the 2009 pandemic | PLoS ONE | c |
| Guvenel et al. 2020^57^ | Epitope-specific airway-resident CD4+ T cell dynamics during experimental human RSV infection | Journal of Clinical Investigation | a |
| Haigh et al. 2019^58^ | A service evaluation of simultaneous near-patient testing for influenza, respiratory syncytial virus, Clostridium difficile and norovirus in a UK district general hospital | Journal of Hospital Infection | d |
| Harcourt et al. 2019^59^ | Developing influenza and respiratory syncytial virus activity thresholds for syndromic surveillance in England | Epidemiology and Infection | a |
| Hardelid et al. 2013^60^ | Mortality caused by influenza and respiratory syncytial virus by age group in England and Wales 1999-2010 | Influenza and Other Respiratory Viruses | c |
| Hodgson et al. 2020^61^ | Estimates for quality of life loss due to respiratory syncytial virus | Influenza and Other Respiratory Viruses | b |
| Hynicka et al. 2012^62^ | Prophylaxis and treatment of respiratory syncytial virus in adult immunocompromised patients | Annals of Pharmacotherapy | d |
| Ieven et al. 2018^63^ | Aetiology of lower respiratory tract infection in adults in primary care: A prospective study in 11 European countries | Clinical Microbiology and Infection | b |
| Jackson et al. 2020^64^ | Results from the WHO external quality assessment for the respiratory syncytial virus pilot, 2016-17 | Influenza and Other Respiratory Viruses | a |
| Jansen et al. 2016^65^ | Prognosis of Good syndrome: Mortality and morbidity of thymoma associated immunodeficiency in perspective | Clinical Immunology | b |
| Johnson et al. 2021^66^ | Hospital utilization rates for influenza and RSV: A novel approach and critical assessment | Population Health Metrics | a |
| Kim et al. 2014^67^ | Respiratory syncytial virus in hematopoietic cell transplant recipients: Factors determining progression to lower respiratory tract disease | Journal of Infectious Diseases | b |
| Kirolos et al. 2019^68^ | A landscape review of the published research output relating to respiratory syncytial virus (RSV) in North & Central America and Europe between 2011-2015 | Journal of Global health | d |
| Kombe et al. 2019^69^ | Model-based estimates of transmission of respiratory syncytial virus within households | Epidemics | a |
| Lam et al. 2019^70^ | Comparative global epidemiology of influenza, respiratory syncytial and parainfluenza viruses, 2010-2015 | Journal of Infection | c |
| Lansbury et al. 2020^71^ | Co-infections in people with COVID-19: A systematic review and meta-analysis | Journal of Infection | b |
| Lee et al. 2015^72^ | High viral load and respiratory failure in adults hospitalized for respiratory syncytial virus infections | Journal of Infectious Diseases | b |
| Li et al. 2018^73^ | Association of seasonal viral acute respiratory infection with pneumococcal disease: A systematic review of population-based studies | BMJ Open | d |
| Li et al. 2019^74^ | Global patterns in monthly activity of influenza virus, respiratory syncytial virus, parainfluenza virus, and metapneumovirus: A systematic analysis | The Lancet Global Health | d |
| Li et al. 2021^75^ | The impact of the 2009 influenza pandemic on the seasonality of human respiratory syncytial virus: A systematic analysis | Influenza and Other Respiratory Viruses | a |
| Ma et al. 2018^76^ | Can we distinguish respiratory viral infections based on clinical features? A prospective pediatric cohort compared to systematic literature review | Reviews in Medical Virology | a |
| Mahony et al. 2013^77^ | Development of a sensitive loop-mediated isothermal amplification assay that provides specimen-to-result diagnosis of respiratory syncytial virus infection in 30 minutes | Journal of Clinical Microbiology | a |
| Mandelia et al. 2020^78^ | Optimal timing of repeat multiplex molecular testing for respiratory viruses | Journal of Clinical Microbiology | b |
| Manikam et al. 2016^79^ | Limited evidence on the management of respiratory tract infections in down's syndrome: A systematic review | Pediatric Infectious Disease Journal | a |
| Mann et al. 2020^80^ | Late breaking abstract – Experimental respiratory syncytial virus infection in adults 60-75 years | European Respiratory Journal. Conference: European Respiratory Society International Congress, ERS | b |
| Matias et al. 2016^81^ | Modelling estimates of age-specific influenza-related hospitalisation and mortality in the United Kingdom | BMC Public Health | c |
| McNally et al. 2014^82^ | Vitamin D receptor (VDR) polymorphisms and severe RSV bronchiolitis: A systematic review and meta-analysis | Pediatric Pulmonology | d |
| Miller et al. 2011^83^ | CT of viral lower respiratory tract infections in adults: Comparison among viral organisms and between viral and bacterial infections | American Journal of Roentgenology | b |
| Millership et al. 2015^84^ | Oseltamivir in influenza outbreaks in care homes: Challenges and benefits of use in the real world | Journal of Hospital Infection | c |
| Mohammed et al. 2016^85^ | A preliminary assessment of the role of ambient nitric oxide exposure in hospitalization with respiratory syncytial virus bronchiolitis | International Journal of Environmental Research and Public Health | a |
| Mollers et al. 2019^86^ | Current practices for respiratory syncytial virus surveillance across the EU/EEA Member States, 2017 | Eurosurveillance | a |
| Moore et al. 2015^87^ | The use of coroner's autopsy reports to validate the use of targeted swabbing rather than tissue collection for rapid confirmation of virological causes of sudden death in the community | Journal of Clinical Virology | d |
| Nicoli et al. 2013^88^ | Influenza and RSV make a modest contribution to invasive pneumococcal disease incidence in the UK | Journal of Infection | c |
| Nicholson et al. 2014^89^ | Randomised controlled trial and health economic evaluation of the impact of diagnostic testing for influenza, respiratory syncytial virus and Streptococcus pneumoniae infection on the management of acute admissions in the elderly and high-risk 18- to 64-year-olds | Health Technology Assessment (Winchester, England) | d |
| Obando-Pacheco et al. 2018^90^ | Respiratory syncytial virus seasonality: A global overview | Journal of Infectious Diseases | c |
| Olchanski et al. 2018^91^ | Palivizumab prophylaxis for respiratory syncytial virus: Examining the evidence around value | Open Forum Infectious Diseases | a |
| Osborne et al. 2021^92^ | PIN70 Psychometric evaluation of the Respiratory Infection Intensity and Impact Questionnaire Version 2 (RiiQATMv2) symptom scores for respiratory syncytial virus | Value in Health | b |
| Paba et al. 2014^93^ | Screening of respiratory pathogens by Respiratory Multi Well System (MWS) r-geneTM assay in hospitalized patients | New Microbiologica | b |
| Pando et al. 2021^94^ | Diversity in the circulation of influenza A(H3n2) viruses in the northern hemisphere in the 2018-19 season | Vaccines | b |
| Papadopoulos et al. 2011^95^ | Viruses and bacteria in acute asthma exacerbations - A GA^2^ LEN-DARE* systematic review | Allergy: European Journal of Allergy and Clinical Immunology | d |
| Permpalung et al. 2019^96^ | Clinical characteristics and treatment outcomes among respiratory syncytial virus (RSV)-infected hematologic malignancy and hematopoietic stem cell transplant recipients receiving palivizumab | Leukemia and Lymphoma | b |
| Price et al. 2019^97^ | Association between viral seasonality and meteorological factors | Scientific Reports | c |
| Richardson et al. 2016^98^ | Comparison of respiratory virus shedding by conventional and molecular testing methods in patients with haematological malignancy | Clinical Microbiology and Infection | b |
| Rose et al. 2015^99^ | Gene expression profiles link respiratory viral infection, platelet response to aspirin, and acute myocardial infarction | PLoS ONE | a |
| Russell et al. 2017^100^ | The human immune response to respiratory syncytial virus infection | Clinical Microbiology Reviews | a |
| Shi et al. 2020^101^ | The etiological role of common respiratory viruses in acute respiratory infections in older adults: A systematic review and meta-analysis | Journal of Infectious Diseases | d |
| Shi et al. 2020^102^ | Global disease burden estimates of respiratory syncytial virus-associated acute respiratory infection in older adults in 2015: A systematic review and meta-analysis | The Journal of infectious diseases | d |
| Shi et al. 2022^103^ | Disease burden estimates of respiratory syncytial virus related acute respiratory infections in adults with comorbidity: A systematic review and meta-analysis | Journal of Infectious Diseases | d |
| Smit et al. 2011^104^ | Adult outpatient experience of the 2009 H1N1 pandemic: Clinical course, pathogens, and evaluation of case definitions | Journal of Infection | b |
| Smith et al. 2017^105^ | Retrospective observational study of atypical winter respiratory illness season using real-time syndromic surveillance, England, 2014-15 | Emerging Infectious Diseases | a |
| Steensels et al. 2017^106^ | Performance evaluation of direct fluorescent antibody, Focus Diagnostics SimplexaTM Flu A/B & RSV and multi-parameter customized respiratory Taqman array card in immunocompromised patients | Journal of Virological Methods | b |
| Sundaram et al. 2014^107^ | Medically attended respiratory syncytial virus infections in adults aged >=50 years: Clinical characteristics and outcomes | Clinical Infectious Diseases | b |
| Sundell et al. 2016^108^ | A four year seasonal survey of the relationship between outdoor climate and epidemiology of viral respiratory tract infections in a temperate climate | Journal of Clinical Virology | a |
| Szabo et al. 2013^109^ | Elevated risk of asthma after hospitalization for respiratory syncytial virus infection in infancy | Paediatric Respiratory Reviews | d |
| Tang et al. 2019^110^ | Impact of a poorly performing point-of-care test during the 2017-2018 influenza season | Journal of Infection | a |
| Tanner et al. 2020^111^ | Covid-19 susceptibility and severity might be modified by vitamin D status: Theoretical and practical considerations | Current Respiratory Medicine Reviews | d |
| Taplitz et al. 2020^112^ | Incidence and impact of community respiratory viral infection (CRV) in haploidentical and matched sibling donors receiving post-transplant cyclophosphamide (PTCy): A CIBMTR analysis | Biology of Blood and Marrow Transplantation | a |
| Thorburn et al. 2015^113^ | The use of next generation sequencing in the diagnosis and typing of respiratory infections | Journal of Clinical Virology | a |
| Thornton et al. 2019^114^ | Assessing the potential of upper respiratory tract point-of-care testing: A systematic review of the prognostic significance of upper respiratory tract microbes | Clinical Microbiology and Infection | b |
| Todkill et al. 2017^115^ | Utility of ambulance data for real-time syndromic surveillance: A pilot in the West Midlands Region, United Kingdom | Prehospital and Disaster Medicine | d |
| Treskova et al. 2021^116^ | Assessment of the effects of active immunisation against respiratory syncytial virus (RSV) using decision-analytic models: A systematic review with a focus on vaccination strategies, modelling methods and input data | PharmacoEconomics | d |
| van Summeren et al. 2021^117^ | Low levels of respiratory syncytial virus activity in Europe during the 2020/21 season: What can we expect in the coming summer and autumn/winter? | Euro Surveillance: Bulletin Europeen sur les Maladies Transmissibles = European Communicable Disease Bulletin | d |
| Verbakel et al. 2020^118^ | Performance and ease of use of a molecular point-of-care test for influenza A/B and RSV in patients presenting to primary care | European Journal of Clinical Microbiology and Infectious Diseases | a |
| Visconti et al. 2017^119^ | Antibody response to paramyxoviruses in Paget's disease of bone | Calcified Tissue International | d |
| Vos et al. 2021^120^ | Lower respiratory tract infection in the community: Associations between viral aetiology and illness course | Clinical Microbiology and Infection | c |
| Waghmare et al. 2013^121^ | Respiratory syncytial virus lower respiratory disease in hematopoietic cell transplant recipients: Viral RNA detection in blood, antiviral treatment, and clinical outcomes | Clinical Infectious Diseases | b |
| Walsh et al. 2013^122^ | Viral shedding and immune responses to respiratory syncytial virus infection in older adults | Journal of Infectious Diseases | b |
| Walsh et al. 2013^123^ | Clinical impact of human coronaviruses 229E and OC43 infection in diverse adult populations | Journal of Infectious Diseases | b |
| Wang et al. 2021^124^ | Global hospital admissions and in-hospital mortality associated with all-cause and virus-specific acute lower respiratory infections in children and adolescents aged 5-19 years between 1995 and 2019: A systematic review and modelling study | BMJ Global Health | a |
| Waterlow et al. 2021^125^ | Competition between RSV and influenza: Limits of modelling inference from surveillance data | Epidemics | d |
| Wilcox et al. 2019^126^ | Attitudes of pregnant women and healthcare professionals toward clinical trials and routine implementation of antenatal vaccination against respiratory syncytial virus: A multicenter questionnaire study | Pediatric Infectious Disease Journal | a |
| Wiseman et al. 2019^127^ | Respiratory syncytial virus (RSV) detection is associated with an increased inflammatory response in stable (non-exacerbating) chronic obstructive pulmonary disease (COPD) patients | American Journal of Respiratory and Critical Care Medicine. Conference | a |
| Wiseman et al. 2020^128^ | Immunological and inflammatory biomarkers of susceptibility and severity in adult respiratory syncytial virus infections | Journal of Infectious Diseases | d |
| Wong et al. 2014^129^ | Initial radiographic features as outcome predictor of adult respiratory syncytial virus respiratory tract infection | AJR. American Journal of Roentgenology | b |
| Xu et al. 2021^130^ | Nanopore metagenomic sequencing of influenza virus directly from respiratory samples: Diagnosis, drug resistance and nosocomial transmission, United Kingdom, 2018/19 influenza season | Eurosurveillance | a |
| Zeevat et al. 2019^131^ | PIN25 Exploratory analysis of the economically justifiable price of an RSV vaccine for the elderly in the Netherlands and the United Kingdom | Value in Health | d |
| Zeevat et al. 2022^132^ | Exploratory analysis of the economically justifiable price of a hypothetical RSV vaccine for older adults in the Netherlands and the United Kingdom | Journal of Infectious Diseases | d |
| Zwaans et al. 2014^133^ | The relevance of respiratory viral infections in the exacerbations of chronic obstructive pulmonary disease – A systematic review | Journal of Clinical Virology | d |

**Footnotes:** a: Study does not present original research in the English language and/or does not include a population of human patients with RSV; b: Study is not conducted in the UK; c: Study does not report an outcome of interest; d: Study does not have an observational design.

Table S12. List of studies excluded at the full-text stage review in the SLR update

| **Authors** | **Title** | **Journal** | **Reason for Exclusion** |
| --- | --- | --- | --- |
| Baird et al. 2022^134^ | Validation of the NeuMoDxTM SARS-CoV-2 Assay with COPAN eNAT and E&O Viral PCR Sample Solution (VPSS) collection media types in comparison with other validated SARS-CoV-2 RNA assays | International Journal of Infectious Diseases : IJID : Official Publication of the International Society for Infectious Diseases | b |
| Bernstein et al. 2022^135^ | Diagnostic accuracy of commercially available tests for respiratory syncytial virus: A scoping literature review in the COVID-19 era | medRxiv | d |
| Bernstein et al. 2022^136^ | Summarizing study characteristics and diagnostic performance of commercially available tests for respiratory syncytial virus: A scoping literature review in the COVID-19 era | The Journal of Applied Laboratory Medicine | d |
| Campbell et al. 2022^137^ | Respiratory viral infections in otherwise healthy humans with inherited IRF7 deficiency | Journal of Experimental Medicine | b |
| Chapman et al. 2021^138^ | Another aspect of COVID pandemic: Where has all the flu gone? | European Respiratory Journal. Conference: International Congress of the European Respiratory Society, ERS | a |
| Chellapuri et al. 2022^139^ | Human parainfluenza 2 & 4: Clinical and genetic epidemiology in the UK, 2013-2017, reveals distinct disease features and co-circulating genomic subtypes | Influenza and Other Respiratory Viruses | c |
| Chellapuri et al. 2022^140^ | Human Parainfluenza 2 & 4: Clinical and genetic epidemiology in the UK, 2013-2017, reveals distinct disease features and co-circulating genomic subtypes | medRxiv | c |
| Falsey et al. 2021^141^ | Risk factors and medical resource utilization of respiratory syncytial virus, human metapneumovirus, and influenza-related hospitalizations in adults – A global study during the 2017-2019 epidemic seasons (Hospitalized Acute Respiratory Tract Infection HARTI Study) | Open Forum Infectious Diseases | b |
| Flynn et al. 2021^142^ | Nasopharyngeal swabs vs. nasal aspirates for respiratory virus detection: A systematic review | Pathogens | d |
| Jiang et al. 2022^143^ | Development of a diagnostic assay by three-tube multiplex real-time PCR for simultaneous detection of nine microorganisms causing acute respiratory infections | Scientific Reports | b |
| Korsten et al. 2021^144^ | World Health Organization influenza-like illness underestimates the burden of respiratory syncytial virus infection in community-dwelling older adults | The Journal of Infectious Diseases | b |
| Krauer et al. 2022^145^ | Estimating RSV seasonality from pandemic disruptions: A modelling study | medRxiv | b |
| Li et al. 2022^146^ | Seasonality of respiratory syncytial virus and its association with meteorological factors in 13 European countries, week 40 2010 to week 39 2019 | Euro Surveillance: Bulletin Europeen sur les Maladies Transmissibles = European Communicable Disease Bulletin | c |
| Li et al. 2022^147^ | Understanding the potential drivers for respiratory syncytial virus rebound during the coronavirus disease 2019 pandemic | Journal of Infectious Diseases | c |
| Lin et al. 2021^148^ | Publisher correction: Distinct patterns of within-host virus populations between two subgroups of human respiratory syncytial virus (Nature Communications, (2021), 12, 1, (5125), 10.1038/s41467-021-25265-4) | Nature Communications | c |
| Magron et al. 2022^149^ | Guides et normes: Usage optimal des immunoglobulines en transplantation d’organes solides |  | b |
| Matheeussen et al. 2021^150^ | Diagnostic performance of the IdyllaTM respiratory panel for molecular detection of influenza A/B in patients presenting to primary care with influenza-like illness during 3 consecutive influenza seasons | Journal of Clinical Virology | b |
| Mulenga et al. 2021^151^ | Longitudinal dynamics of a blood transcriptomic signature of tuberculosis | American Journal of Respiratory and Critical Care Medicine | b |
| Popowitch et al. 2022^152^ | Comparative performance of the Luminex NxTAG Respiratory Pathogen Panel, GenMark eSensor Respiratory Viral Panel, and BioFire FilmArray Respiratory Panel | Microbiology Spectrum | b |
| Shaterian et al. 2021^153^ | Facemask and respirator in reducing the spread of respiratory viruses; A systematic review | Archives of Academic Emergency Medicine | b |
| Stewart et al. 2022^154^ | Pandemic, epidemic, endemic: B Cell repertoire analysis reveals unique anti-viral responses to SARS-CoV-2, Ebola and respiratory syncytial virus | Frontiers in Immunology | a |

**Footnotes:** a: Study does not present original research in the English language and/or does not include a population of human patients with RSV; b: Study is not conducted in the UK; c: Study does not report an outcome of interest; d: Study does not have an observational design.

Appendix S4. PRISMA Diagram for the Original SLR Search


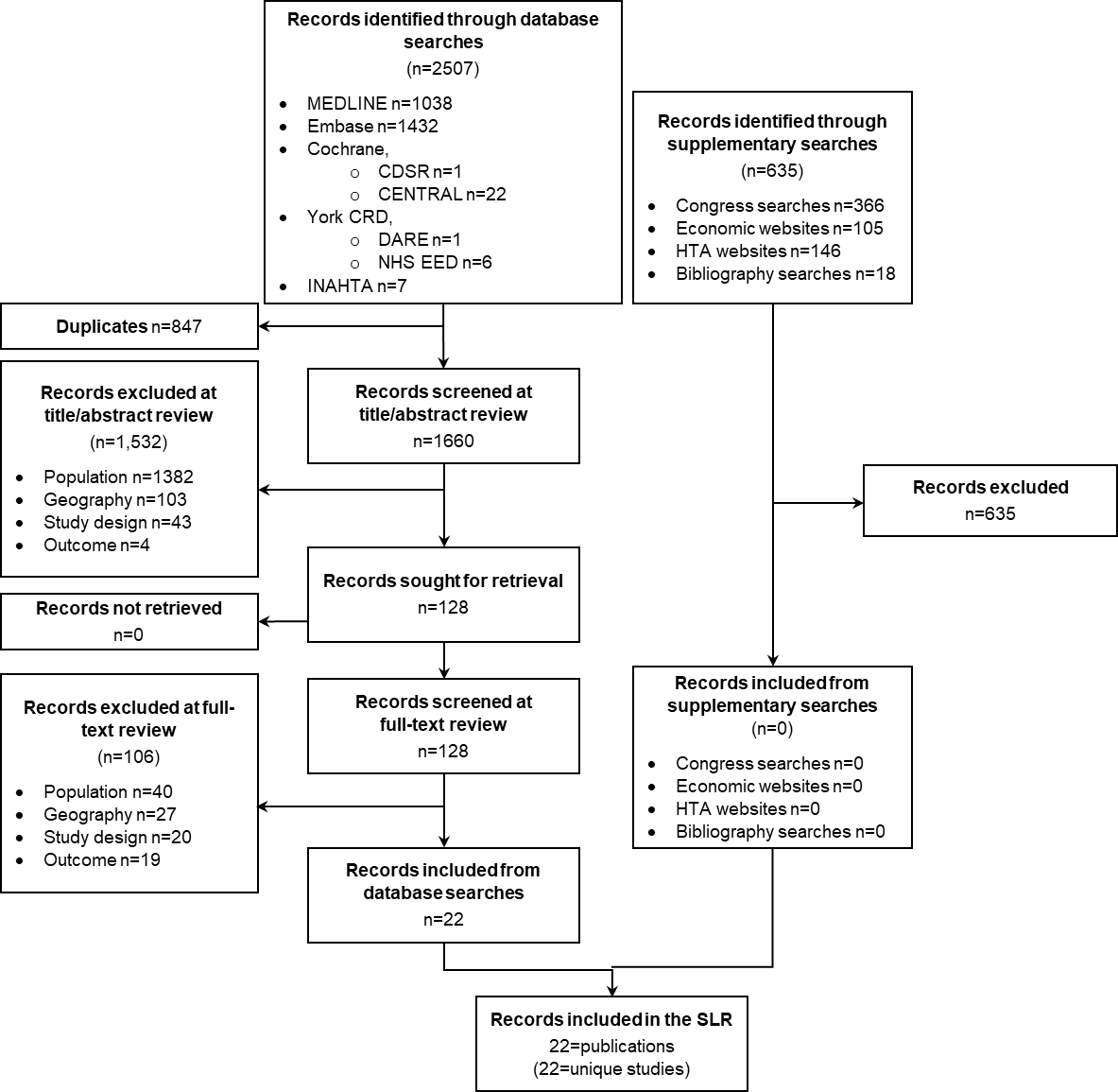


**Abbreviations:** CRD, Centre for Reviews and Dissemination; HTA, Heath Technology Assessment; INAHTA, International Heath Technology Assessment Database; PRISMA, Preferred Reporting Items for Systematic Reviews and Meta-Analyses.

Appendix S5. Characteristics of Studies Included in the SLR and Extracted Results

Table S13. Characteristics and reported outcomes of studies reporting RSV burden in a general population and/or with a respiratory indicator

| **Study** | **Years and Period of Data Collection** | **Study Design; Method of Data Reporting** | **Country** | **Source of Data** | **N** | **Method of RSV Detection** | **RSV Sampling Methods** | **Age, years** | **Outcomes** |  |
| --- | --- | --- | --- | --- | --- | --- | --- | --- | --- | --- |
| **Berry 2020^2^** | 2018–2019, seasonal | Prospective cohort; measured | England | Single centre; Respiratory Assessment Unit at a large teaching hospital | 390 (PCR testing); 755 (POC testing) | POC testing (antigen); multiplex PCR | Pharyngeal swabs | >16 | Incidence 1.6% (12/755) for POC testing; 7.4% (29/390) for PCR testing in adults admitted to respiratory unit with a suspected respiratory tract infection; median Charlson co-morbidity index scores reported |  |
| **Dadhwal 2022^5^** | 2019–2020, annual | Retrospective cohort; measured | England | Single centre; the ICU at a large tertiary referral centre | 93 (COVID-19 group); 53 (other viral pneumonia group) | PCR | NR | >18 | Incidence 15.1% (8/53) in patients admitted to ICU with non-COVID-19 viral pneumonia |  |
| **Fleming 2015^6^** | 1995–2009, seasonal | National registry/database; modelled | UK | CPRD database (GP episodes); HES database (hospitalisations) | Adult patients in the ONS database; exact value NR | NR | NR | 18–49, 50–64 | 487,247 GP episodes, 17,799 hospitalisations and 8,482 deaths attributable to RSV per average season (general UK population ≥18 years). Mean seasonal RSV-attributable antibiotic prescriptions were reported as 159,223 (18–49 years) and 149,229 (50–64 years). RSV-attributable outcomes (GP episodes, hospitalisations and deaths) were reported by age group and by risk groups, defined according to the chronic conditions indicative of risk for severe influenza as per UK recommendation for influenza vaccination |  |
| **Green 2013^155^** | 2009–2011, seasonal | National registry/database; modelled | England | Multicentre; samples tested for respiratory viruses in England from the English Respiratory Datamart system | NR | Positive RSV sample number per week on English Respiratory Datamart system | NR | 15–44, ≥45 | Incidence 6.01% (95% CI 4.68–6.85) (15–44 years, 2010), 7.62% (95% CI 6.36–8.67) (≥45 years, 2010), 2.21% (95% CI 1.79–2.49) (15–44 years, 2011), 6.12% (95% CI 5.42–6.61) (≥45 years, 2011) |  |
| **Hodgson 2020^11^** | 2010–2017, annual | National registry/database; measured | England | Multicentre; hospital laboratories; inpatients, outpatients, primary care settings and patients in the community were included | NR | NR | NR | 15–24; 25–44; 45–64 | Incidence 0.784 (15–24 years), 1.19 (25–44 years), 2.42 (45–64 years) per 100,000 per year |  |
| **Hughes 2016^12^** | 2011–2013, seasonal | National registry/database; measured | England | Multicentre; emergency departments | NR | NR | NR | ≥18 | Data on number of RSV isolates detected weekly presented in figure (not digitised) |  |
| **Johannesen 2022^14^** | 2006–2018, annual | National registry/database; modelled | England, Scotland | Multicentre; hospital admissions and registries/laboratories | NR | NR | NR | 18–64 | Estimated incidence of RSV 0.1 (18–64 years, England) and NR (18–64 years, Scotland) per 1,000 per year in respiratory infection patients admitted to hospital; estimated incidence also reported for acute upper respiratory tract infection, pneumonia and influenza, bronchitis and bronchiolitis, and unspecified lower respiratory tract infections patients admitted to hospital; weekly number of RSV-associated hospitalisations for different age groups provided graphically but not digitised |  |
| **Morbey 2017^16^** | 2013–2015, annual | National registry/database; modelled | England | Syndromic and laboratory data retrieved from NHS 111 and SGSS | All adult patients who called NHS 111 between September 2013–July 2015 (exact N NR) | NR | NR | 15–64 | 131 calls to NHS 111 on average per week associated with RSV (15–64 years) |  |
| **Morbey 2018^17^** | 2011–2015, annual | National registry/database; modelled | England | PHE GP in-hours and out-of-hours syndromic surveillance systems | Modelled cohort of 10,000 patients | NR | NR | 15–64 | 2.18 average weekly GP consultations associated with RSV (15–64 years) |  |
| **Morris 2021^18^** | 2019–2020, seasonal | Prospective cohort; measured | England | Single centre; hospital laboratory | 642 (including 92 children) | Xpress Flu/RSV test and the AusDiagnostics Respiratory Viruses 16‐well Assay | Nose, throat, and combined nose and throat swabs, as well as nasopharyngeal aspirate | ≥18 | 39 RSV cases in adult patients with samples collected for influenza/RSV testing |  |
| **Partridge 2022^19^** | 2015–2020, annual | Mixed-methods study (prospective and retrospective); measured and modelled | England | Single centre; haematology department | 412 pre-universal mask use (2015–18); 138 post-universal mask use (2019–20) | PCR assay | Nose and/or throat swabs, sputum or bronchoalveolar lavage | ≥18 | RSV cases per total respiratory viral infections from community: 597/9,076 (2015–18), 594/4,546 (2019–20) |  |
| **Ponsford 2021^20^** | 2015–2018, annual | Prospective case control/matched; measured | Wales | Single centre; specialist immunodeficiency centre in a hospital | 44 (PAD patients); 42 (healthy controls) | NxTAG® Respiratory Pathogen Panel | “Acute” nasal swabs | ≥18 | Incidence 0.3% (3/626) in otherwise healthy population; -0.143 change in BIPAD-Q symptom score from baseline in 7 days after RSV detection |  |
| **Poole 2020^21^** | 2020, seasonal | Prospective cohort; measured | England | Multicentre; emergency department or acute medical unit of Southampton General Hospital (hospital and tertiary referral centre) and Royal Hampshire Hospital | 727 | Molecular respiratory virus panel | Combined nose and throat swab | ≥18 | 29 RSV cases per 533 patients with non SARS-CoV-2 PCR-detected respiratory virus infection (or per 727 patients with any PCR-detected respiratory infection); RSV incidence in PCR-confirmed respiratory virus patient population provided per year: 0.9% (2015), 3.4% (2016), 1.8% (2018), 1.2% (2019), 0.09% (2020) |  |
| **RESCEU^15^** | 2017–2019, seasonal | Prospective cohort; measured | England | Multicentre; GP practices | 349 (UK population) | Xpress Flu/RSV assay | Nasopharyngeal sample | ≥60 | RSV cases in all patients enrolled in RESCEU study in England: 8/175 (2018), 10/174 (2019), 18/349 (2018 and 2019 total) |  |
| **Tanner 2012^25^** | 2009–2010, seasonal | Retrospective cohort; measured | UK | Multicentre; hospitals and GPs in the West Midlands region of the UK | 4,821 | RSV detection using real time PCR | Nasopharyngeal aspirates, nose and throat swabs, and bronchoalveolar lavages | 15–24; 25–44; 45–64 | Incidence 10.3% (15–24 years), 10.2% (25–44 years), 17.9% (45–64 years) |  |
| **Youngs 2019^27^** | 2018, seasonal | Retrospective cohort; measured | England | Single centre emergency department | 672 | RSV as detected by PCR test | Throat swab | ≥18 | RSV cases in all adults admitted to emergency department with possible influenza: 9/672 |  |
| **Zhao 2014^28^** | 2009–2012, annual | National registry/database; measured | England | Multicentre; Hospital laboratories; inpatients, outpatients, primary care settings and patients in the community were included | 43,949 | NR | Nasopharyngeal aspirate, tracheal secretion and nasal and throat swab (most common sample types) | 15–44; 45–64 | RSV incidence data presented in figure; not digitised |  |

**Abbreviations:** BIPAD-Q: Burden of Infection as measured by Primary Antibody Deficiency; CAP: community-acquired pneumonia; COVID-19: coronavirus disease 2019; CPRD: Clinical Practice Research Datalink; ELISA: enzyme linked immunosorbent assay; GP: general practice; HES: Hospital Episode Statistics; HSCT: haematopoietic stem cell transplantation; ICU: intensive care unit; IgM: Immunoglobulin M; ILI: influenza-like illnesses; IPD: invasive pneumococcal disease; MI: myocardial infarction; NR: not reported; ONS: Office for National Statistics; PAD: primary antibody deficiency; PCR: polymerase chain reaction; PHE: Public Health England; POC: Point of contact; RSV: respiratory syncytial virus; SGSS: Second Generation Surveillance System; UK: United Kingdom.

Table S14. Characteristics and reported outcomes of studies reporting RSV burden in an elderly population

| **Study** | **Years and Period of Data Collection** | **Study Design; Method of Data Reporting** | **Country** | **Source of Data** | **N** | **Method of RSV Detection** | **RSV Sampling Methods** | **Age, years** | **Outcomes** |
| --- | --- | --- | --- | --- | --- | --- | --- | --- | --- |
| **Blackburn 2018^3^** | 2004–2015, annual | National registry/database; modelled | England | Multicentre; hospital admissions for MI and stroke; All positive respiratory virus test results reported by hospital laboratories in England | NR | RSV detection in samples submitted to LabBase from primary and secondary healthcare settings | NR | 65–74; ≥75 | RSV incidence in elderly patients with MI: 1.4% (65–74 years, 2004–2015), 1.6% (≥75 years, 2004–2015), 2.0% (65–74 years, 2010–2015), 1.7% (≥75 years, 2010–2015); in elderly patients with stroke: 0.7% (≥75 years, 2004–2015), 0.9% (≥75 years, 2010–2015); in elderly patients with ischaemic stroke: 1.0% (≥75 years, 2004–2015), 1.2% (≥75 years, 2010–2015) |
| **Chiavenna 2019^4^** | 2009–2017, annual | National registry/database; modelled | England | Multicentre; GP practices and diagnostic laboratories | 45,601 | RSV confirmed in ILI cases by PHE reference laboratory or RSV count extracted from the SGSS database | NR | ≥65 | RSV incidence 3.91% in invasive pneumococcal disease patients (95% CI 1.83–6.38) (≥65 years) |
| **Fleming 2015^6^** | 1995–2009, seasonal | National registry/database; modelled | UK | CPRD database (GP episodes); HES database (hospitalisations) | Adult patients in the ONS database; exact value NR | NR | NR | ≥65; 65-74;  ≥75 | 175,070 GP episodes, 14,039 hospitalisations and 7,915 deaths attributable to RSV per average season in the elderly (≥65 years), corresponding to 36% of all GP episodes, 79% of hospitalisations and 93% of RSV-attributable deaths in all adults (≥18 years). Mean seasonal RSV-attributable antibiotic prescriptions were reported as 98,921 (65–74 years) and 91,535 (≥75 years). RSV-attributable outcomes (GP episodes, hospitalisations and deaths) were reported by age group and by risk groups, defined according to the chronic conditions indicative of risk for severe influenza as per UK recommendation for influenza vaccination |
| **Hodgson 2020^11^** | 2010–2017, annual | National registry/database; modelled | England | Multicentre; hospital laboratories; inpatients, outpatients, primary care settings and patients in the community were included | NR | NR | NR | ≥65 | RSV incidence 2.85 per 100,000 per year (≥65 years) |
| **Johannesen 2022^14^** | 2006–2018, annual | National registry/database; modelled | England, Scotland | Multicentre; hospital admissions and registries/laboratories | NR | NR | NR | 65–74; 75–84; ≥85 | Estimated RSV incidence 0.9 (65–74 years, England), 2.8 (75–84 years, England), 6.0 (≥85 years, England), 0.6 (65–74 years, Scotland), 3.0 (75–84 years, England), 5.0 (≥85 years, England) per 1,000 per year in respiratory infection patients admitted to hospital; estimated incidence also reported for acute upper respiratory tract infection, pneumonia and influenza, bronchitis and bronchiolitis, and unspecified lower respiratory tract infections patients admitted to hospital; weekly number of RSV-associated hospitalisations for different age groups provided graphically but not digitised |
| **Morbey 2017^16^** | 2013–2015, annual | National registry/database; modelled | England | Syndromic and laboratory data retrieved from NHS 111 and SGSS | All adult patients who called NHS 111 between September 2013–July 2015 (exact N NR) | NR | NR | ≥65 | 54 calls to NHS 111 on average per week associated with RSV (≥65 years) |
| **Morbey 2018^17^** | 2011–2015, annual | National registry/database; modelled | England | PHE GP in-hours and out-of-hours syndromic surveillance systems | Modelled cohort of 10,000 patients | NR | NR | ≥65 | 0.771 average weekly GP consultations associated with RSV (≥65 years) |
| **Sharp 2022^23^** | 2010–2017, seasonal | National registry/database; modelled | England | Multicentre; UK laboratory data using the SGSS and hospital data from the HES database | 4,254,927 hospital admissions | NR | NR | 65–74; ≥75 | RSV-associated respiratory hospitalisations estimated as 71 (95% CI 52–90) (65–74 years) and 251 (95% CI 186–316) (≥75 years) per 100,000, seasonal annual average. RSV-attributable admission rate by age group also presented by season, and mean seasonal admissions were reported by diagnosis and age group. Weekly admissions attributed to RSV presented graphically but not digitised |
| **Tanner 2012^25^** | 2009–2010, seasonal | Retrospective cohort; measured | UK | Multicentre; hospitals and GPs in the West Midlands region of the UK | 4,821 | RSV detection using real time PCR | Nasopharyngeal aspirates, nose and throat swabs, and bronchoalveolar lavages | ≥65 | Incidence 26.5% (≥65 years) |
| **Zhao 2014^28^** | 2009–2012, annual | National registry/database; measured | England | Multicentre; Hospital laboratories; inpatients, outpatients, primary care settings and patients in the community were included | 43,949 | NR | Nasopharyngeal aspirate, tracheal secretion and nasal and throat swab (most common sample types) | ≥65 | RSV incidence data presented in figure; not digitised |

**Abbreviations:** CAP: community-acquired pneumonia; CPRD: Clinical Practice Research Datalink; ELISA: enzyme linked immunosorbent assay; GP: general practice; HES: Hospital Episode Statistics; HSCT: haematopoietic stem cell transplantation; IgM: Immunoglobulin M; ILI: influenza-like illnesses; IPD: invasive pneumococcal disease; MI: myocardial infarction; NR: not reported; ONS: Office for National Statistics; PAD: primary antibody deficiency; PCR: polymerase chain reaction; PHE: Public Health England; POC: Point of contact; RSV: respiratory syncytial virus; SGSS: Second Generation Surveillance System; UK: United Kingdom.

Table S15. Characteristics and reported outcomes of studies reporting RSV burden in a co-morbid population

| **Study** | **Years and Period of Data Collection** | **Study Design; Method of Data Reporting** | **Country** | **Source of Data** | **N** | **Method of RSV Detection** | **RSV Sampling Methods** | **Age, years** | **Outcomes** |
| --- | --- | --- | --- | --- | --- | --- | --- | --- | --- |
| **Blackburn 2018^3^** | 2004–2015, annual | National registry/database; modelled | England | Multicentre; hospital admissions for MI and stroke; All positive respiratory virus test results reported by hospital laboratories in England | NR | RSV detection in samples submitted to LabBase from primary and secondary healthcare settings | NR | 65–74; ≥75 | RSV incidence in elderly patients with MI: 1.4% (65–74 years, 2004–2015), 1.6% (≥75 years, 2004–2015), 2.0% (65–74 years, 2010–2015), 1.7% (≥75 years, 2010–2015); in elderly patients with stroke: 0.7% (≥75 years, 2004–2015), 0.9% (≥75 years, 2010–2015); in elderly patients with ischaemic stroke: 1.0% (≥75 years, 2004–2015), 1.2% (≥75 years, 2010–2015) |
| **Flight 2014^7^** | 2010–NR, annual | Prospective cohort; measured | England | Single centre; patients attending the Manchester Adult Cystic Fibrosis Centre | 98 | PCR assay | Sputum, nose swabs and throat swabs | ≥18 | RSV incidence 2% in cystic fibrosis patients with respiratory viruses (0.04 cases/patient-year, 95% CI: 0.001-0.09) |

**Abbreviations:** MI: myocardial infarction; NR: not reported; PCR: polymerase chain reaction; RSV: respiratory syncytial virus.

Table S16. Characteristics and reported outcomes of studies reporting RSV burden in an immunocompromised population

| **Study** | **Years and Period of Data Collection** | **Study Design; Method of Data Reporting** | **Country** | **Source of Data** | **N** | **Method of RSV Detection** | **RSV Sampling Methods** | **Age, years** | **Outcomes** |
| --- | --- | --- | --- | --- | --- | --- | --- | --- | --- |
| **Balassa 2019^1^** | 2015–2017, annual | Retrospective cohort; measured | England | Single centre; hospital | 300 | PCR assay | Throat swabs | ≥18 | 49 RSV respiratory tract infections in 46 patients diagnosed in estimated 300 patients post allo-HSCT over 26 months; of them 23 (46.9%) were upper and 26 (53.1%) were lower respiratory tract infections. 9/49 episodes were considered nosocomial. 1 RSV-attributed death was reported (2.0% of all RSV infections). Treatment setting (including ICU admission) and length of in-patient stay, as well as ribavirin and intravenous immunoglobulin use, were also reported |
| **Gorcea 2017^9^** | 2010–2015, annual | Retrospective cohort; measured | England | Single centre; hospital | 280 | PCR assay | Nose and throat swabs | ≥18 | RSV prevalence was 16% (46 RSV infections in 280 HSCT patients). 13/46 (28%) were identified as contracted in the hospital. 23 RSV cases were analysed, in which 1 RSV-associated death was reported. At diagnosis, 7/23 patients had lower and 16/23 had upper respiratory tract infection. Ribavirin use and treatment duration were also reported |
| **Inkster 2017^13^** | December 2015 (Outbreak 1) and December 2016 (Outbreak 2), seasonal | Prospective cohort study; measured | Scotland | Single centre; haemato-oncology ward | 10 (December 2015); 12 (December 2016) | PCR assay | Saline gargle | ≥18 | Two RSV outbreaks in a haemato-oncology ward were described. 8 cases of RSV were reported in the first outbreak (mortality: 37.5%) and 12 cases in the second (mortality: 8.3%). Ribavirin and immunoglobulin use, as well as symptoms, were also reported |
| **Morris 2021^18^** | 2019–2020, seasonal | Prospective cohort; measured | England | Single centre; hospital laboratory | 642 (including 92 children) | Xpress Flu/RSV test and the AusDiagnostics Respiratory Viruses 16‐well Assay | Nose, throat, and combined nose and throat swabs, as well as nasopharyngeal aspirate | ≥18 | 6 RSV cases in 41 Bone Marrow Transplant Unit patients |
| **Partridge 2022^19^** | 2015–2020, annual | Mixed-methods study (prospective and retrospective); measured and modelled | England | Single centre; haematology department | 412 pre-universal mask use (2015–18); 138 post-universal mask use (2019–20) | PCR assay | Nose and/or throat swabs, sputum or bronchoalveolar lavage | ≥18 | RSV incidence 5/412 (1.21%) in stem cell transplant patients before mask mandate and 5/138 (3.62%) after. Rates were also adjusted for adult population incidence |
| **Ponsford 2021^20^** | 2015–2018, annual | Prospective case control/matched; measured | Wales | Single centre; specialist immunodeficiency centre in a hospital | 44 (PAD patients); 42 ( healthy controls) | NxTAG® Respiratory Pathogen Panel | “Acute” nasal swabs | ≥18 | RSV incidence 7/870 (0.8%) in primary antibody deficiency patients; 0.469 change in BIPAD-Q symptom score from baseline in 7 days after RSV detection |

**Abbreviations:** BIPAD-Q: Burden of Infection as measured by Primary Antibody Deficiency; HSCT: haematopoietic stem cell transplantation; ICU: intensive care unit; PAD: primary antibody deficiency; PCR: polymerase chain reaction; RSV: respiratory syncytial virus.

Table S17. Characteristics and reported outcomes of studies reporting RSV burden in a co-infected population

| **Study** | **Years and Period of Data Collection** | **Study Design; Method of Data Reporting** | **Country** | **Source of Data** | **N** | **Method of RSV Detection** | **RSV Sampling Methods** | **Age, years** | **Outcomes** |
| --- | --- | --- | --- | --- | --- | --- | --- | --- | --- |
| **Chiavenna 2019^4^** | 2009–2017, annual | National registry/database; modelled | England | Multicentre; GP practices and diagnostic laboratories | 45,601 | RSV confirmed in ILI cases by PHE reference laboratory or RSV count extracted from the SGSS database | NR | 15–44; 45–64; ≥65 | RSV incidence 0% (15–44 years), 4.18% (95% CI: 1.58–6.91) (45–64 years), 3.91% (95% CI: 1.83–6.38) (≥65 years) in patients with invasive pneumococcal disease |
| **Gadsby 2016^8^** | 2012–2014, annual | Retrospective cohort; measured | Scotland | Multicentre: tertiary care hospitals | 323 | PCR assay | Sputum (96%) or endotracheal aspirate (4%) specimens | Median: 67 | RSV incidence 4/323 (1.2%) in community-acquired pneumonia patients |
| **Russell 2018^22^** | 2006–2016, annual | Retrospective cohort; measured | Scotland | Multicentre; two tertiary care hospitals | 14 | RSV (virologic testing of throat swabs from leptospirosis patients), IgM ELISA testing | Throat swabs | ≥18;  Median: 26 | RSV incidence 1/8 in leptospirosis patients |
| **Swets 2022^24^** | 2020–2021, annual | Prospective cohort; measured | UK | Multicentre; hospitals | 212,466 | RT-PCR assay | NR | ≥18 | RSV incidence 220/17,011 in COVID-19 patients admitted to hospital. All-cause in-hospital mortality, drug and ventilator use, ICU admissions and inpatient stays were also reported |
| **Weir-Mccall 2022^26^** | 2019–2020, annual | Retrospective cohort; measured | England | Multicentre; centres of the English national ECMO service | 113 | RT-PCR assay | NR | ≥18 | RSV incidence 0/64 (0%) in COVID-19 and 2/49 (4%) in non-COVID-19 viral pneumonia patients admitted for ECMO support |

**Abbreviations:** ECMO: extracorporeal membrane oxygenation; ELISA: enzyme linked immunosorbent assay; ICU: intensive care unit; ILI: influenza-like illnesses; RT-PCR: reverse transcriptase polymerase chain reaction; PHE: Public Health England; RSV: respiratory syncytial virus; SGSS: Second Generation Surveillance System.

Appendix S6. Gap Analysis of Studies Included in the SLR

| **Population** | **Outcome** | **Total Number of Records** | **Specific Outcomes** | **Number of Records** | **Notes** |
| --- | --- | --- | --- | --- | --- |
| **Elderly Patients (≥65 years)** | Economic | 5 | Direct costs and/or resource use in RSV patients | 5 | GPs and hospitalisations (Fleming 2015)^6^, GP visits per 10,000 (Morbey 2018)^17^, NHS 111 calls by patients with a respiratory indicator (Morbey 2017)^16^, hospital admissions (Johannesen 2022 and Sharp 2022)^14, 23^; Fleming 2015 and Sharp 2022 both report hospital admissions for 65–74 and ≥75 age groups; no other studies comparable |
|  |  |  | Indirect costs and/or resource use in RSV patients | 0 | N/A |
|  | Epidemiological | 7 | Incidence or prevalence of RSV | 6 | 6 studies report incidence; all report on non-comparable populations (co-morbidities, co-infections and respiratory indicators) |
|  |  |  | RSV mortality | 1 | Reports all deaths attributable to RSV respiratory disease per season |
|  |  |  | Incidence or prevalence of RSV complications | 0 | N/A |
|  |  |  | Incidence or prevalence of RSV presentation | 0 | N/A |
|  | Clinical | 0 | RSV symptom presence or duration | 0 | N/A |
|  |  |  | Specific RSV symptoms | 0 | N/A |
| **Co-morbid Patients** | Economic | 0 | Direct costs and/or resource use in RSV patients | 0 | N/A |
|  |  |  | Indirect costs and/or resource use in RSV patients | 0 | N/A |
|  | Epidemiological | 2 | Incidence or prevalence of RSV | 2 | Both studies report incidence; similar incidence rate although not directly comparable as patients have different co-morbidities |
|  |  |  | RSV mortality | 0 | N/A |
|  |  |  | Incidence or prevalence of RSV complications | 0 | N/A |
|  |  |  | Incidence or prevalence of RSV presentation | 0 | N/A |
|  | Clinical | 0 | RSV symptom presence or duration | 0 | N/A |
|  |  |  | Specific RSV symptoms | 0 | N/A |
| **Co-infected Patients** | Economic | 1 | Direct costs and/or resource use in RSV patients | 1 | Reports length of hospital stay, ICU admissions, drug use and ventilator use |
|  |  |  | Indirect costs and/or resource use in RSV patients | 0 | N/A |
|  | Epidemiological | 5 | Incidence or prevalence of RSV | 5 | All studies report incidence |
|  |  |  | RSV mortality | 0 | N/A |
|  |  |  | Incidence or prevalence of RSV complications | 0 | N/A |
|  |  |  | Incidence or prevalence of RSV presentation | 0 | N/A |
|  | Clinical | 0 | RSV symptom presence or duration | 0 | N/A |
|  |  |  | Specific RSV symptoms | 0 | N/A |
| **Immunocompromised Patients** | Economic | 3 | Direct costs and/or resource use in RSV patients | 3 | All resource use, including hospital length of stay, drug use and ICU admission |
|  |  |  | Indirect costs and/or resource use in RSV patients | 0 | N/A |
|  | Epidemiological | 6 | Incidence or prevalence of RSV | 6 | All 6 report incidence |
|  |  |  | RSV mortality | 3 | Estimated proportion of RSV-attributable mortality ranged from 0–30% |
|  |  |  | Incidence or prevalence of RSV complications | 0 | N/A |
|  |  |  | Incidence or prevalence of RSV presentation | 2 | Both report URTI/LRTI presentation and are comparable |
|  | Clinical | 2 | RSV symptom presence or duration | 1 | RSV symptoms in an outbreak in a haematology ward, very small sample size (<10 patients) |
|  |  |  | Specific RSV symptoms | 1 | Change in BIPAD-Q score reported |
| **General Patients** | Economic | 0 | Direct costs and/or resource use in RSV patients | 0 | N/A |
|  |  |  | Indirect costs and/or resource use in RSV patients | 0 | N/A |
|  | Epidemiological | 1 | Incidence or prevalence of RSV | 1 | All report incidence |
|  |  |  | RSV mortality | 0 | N/A |
|  |  |  | Incidence or prevalence of RSV complications | 0 | N/A |
|  |  |  | Incidence or prevalence of RSV presentation | 0 | N/A |
|  | Clinical | 1 | RSV symptom presence or duration | 1 | Change in BIPAD-Q score reported |
|  |  |  | Specific RSV symptoms | 1 | N/A |
| **Respiratory Indicator Patients** | Economic | 4 | Direct costs and/or resource use in RSV patients | 4 | GPs and hospitalisations (Fleming 2015)^6^, GP visits per 10,000 (Morbey 2018)^17^, NHS 111 calls by patients with a respiratory indicator (Morbey 2017)^16^, and hospitalisations (Johannesen 2022)^14^; not comparable |
|  |  |  | Indirect costs and/or resource use in RSV patients | 0 | N/A |
|  | Epidemiological | 14 | Incidence or prevalence of RSV | 13 | All report incidence |
|  |  |  | RSV mortality | 1 | Reports all deaths attributable to RSV respiratory disease per season |
|  |  |  | Incidence or prevalence of RSV complications | 0 | N/A |
|  |  |  | Incidence or prevalence of RSV presentation | 0 | N/A |
|  | Clinical | 0 | RSV symptom presence or duration | 0 | N/A |
|  |  |  | Specific RSV symptoms | 0 | N/A |

**Abbreviations:** BIPAD-Q: Burden of Infection in Primary Antibody Deficiency symptom score; GP: general practice; NHS, National Health Service; LRTI: lower respiratory tract infection; RSV: respiratory syncytial virus; URTI: upper respiratory tract infection.

Appendix S7. Quality Assessment of Studies Included in the SLR

A summary of the AHFMR quality assessment checklist results conducted for each study included in the SLR is detailed below. Overall, the majority (n=23) of included studies were considered to have a low risk of bias. A total of five studies had a high risk of bias, measured as reporting high risk for 3 or more domains in the AHFMR checklist.^11, 13, 17, 16, 28^

Overall all 28 studies were considered to report a clear and appropriate study design, describe appropriate analytic methods and present conclusions supported by the study results.^1-9, 11-28, 155^ All but two studies captured in the SLR reported well-defined or robustly measured outcomes (n=26).^1-9, 11-21, 23-28^ The remaining two studies were considered to only partially describe outcome measurement, because viral testing measures were not sufficiently reported.^22, 155^

A large number of the included studies (n=14) did not sufficiently describe the characteristics of patients,^2-4, 6, 11, 12, 14-17, 23, 27, 28, 155^ while four studies were considered to have partially reported characteristics,^1, 19, 21, 25^ and only ten have described characteristics in sufficient detail.^5, 7-9, 13, 18, 20, 22, 24, 26^

Only 17 of the 28 included studies were considered to report an appropriate sample size.^2-4, 6, 11, 12, 14, 16-19, 21, 23-25, 27, 28^ Nine studies were determined to have a partially appropriate sample size, with limiting factors including a narrow study inclusion criteria.^1, 5, 7-9, 15, 20, 26, 155^ Only two studies were considered to have an inappropriate sample size, one of which only assessed 14 patients,^22^ and the other did not report the sample size.^13^

The majority (n=22) of studies included in the SLR did not report whether the study was controlled for confounding.^1, 3-5, 7, 9, 11, 13-19, 21-23, 25-28, 155^ Amongst the five studies which reported how they controlled for confounding, two used linear regression analysis,^8, 12^ one included a control study group,^20^ one weighted the data by age to reflect the UK profile^6^ and one used a multivariable model that adjusted for multiple confounders including age, sex and number of co-morbidities.^24^

Table S18. Summary of study quality as assessed by the AHFMR checklist^156^

| **Study** | **Is the question/objective sufficiently described?** | **Is the study design evident and appropriate?** | **Is the method of subject group selection described and appropriate?** | **Were the subject characteristics sufficiently described?** | **Outcome measure(s) well defined and robust to measurement?** | **Was the sample size appropriate?** | **Were the analytic methods described/justified and appropriate?** | **Was some estimate of variance reported for the main results?** | **Was the study controlled for confounding?** | **Were results reported in sufficient detail?** | **Were conclusions supported by the results?** |
| --- | --- | --- | --- | --- | --- | --- | --- | --- | --- | --- | --- |
| Studies assessing a general/respiratory indicator patient population | | | | | | | | | | | |
| **Berry 2020^2^** | 🔴 | 🔴 | 🔴 | 🔴 | 🔴 | 🔴 | 🔴 | 🔴 | 🔴 | 🔴 | 🔴 |
| **Dadhwal 2022^5^** | 🔴 | 🔴 | 🔴 | 🔴 | 🔴 | 🔴 | 🔴 | 🔴 | 🔴 | 🔴 | 🔴 |
| **Fleming 2015^6^** | 🔴 | 🔴 | 🔴 | 🔴 | 🔴 | 🔴 | 🔴 | 🔴 | 🔴 | 🔴 | 🔴 |
| **Green 2013^155^** | 🔴 | 🔴 | 🔴 | 🔴 | 🔴 | 🔴 | 🔴 | 🔴 | 🔴 | 🔴 | 🔴 |
| **Hodgson 2020^11^** | 🔴 | 🔴 | 🔴 | 🔴 | 🔴 | 🔴 | 🔴 | 🔴 | 🔴 | 🔴 | 🔴 |
| **Hughes 2016^12^** | 🔴 | 🔴 | 🔴 | 🔴 | 🔴 | 🔴 | 🔴 | 🔴 | 🔴 | 🔴 | 🔴 |
| **Johannesen 2022^14^** | 🔴 | 🔴 | 🔴 | 🔴 | 🔴 | 🔴 | 🔴 | 🔴 | 🔴 | 🔴 | 🔴 |
| **Morbey 2018^17^** | 🔴 | 🔴 | 🔴 | 🔴 | 🔴 | 🔴 | 🔴 | 🔴 | 🔴 | 🔴 | 🔴 |
| **Morbey 2017^16^** | 🔴 | 🔴 | 🔴 | 🔴 | 🔴 | 🔴 | 🔴 | 🔴 | 🔴 | 🔴 | 🔴 |
| **Morris 2021^18^** | 🔴 | 🔴 | 🔴 | 🔴 | 🔴 | 🔴 | 🔴 | 🔴 | 🔴 | 🔴 | 🔴 |
| **Partridge 2022^19^** | 🔴 | 🔴 | 🔴 | 🔴 | 🔴 | 🔴 | 🔴 | 🔴 | 🔴 | 🔴 | 🔴 |
| **Ponsford 2021^20^** | 🔴 | 🔴 | 🔴 | 🔴 | 🔴 | 🔴 | 🔴 | 🔴 | 🔴 | 🔴 | 🔴 |
| **Poole 2020^21^** | 🔴 | 🔴 | 🔴 | 🔴 | 🔴 | 🔴 | 🔴 | 🔴 | 🔴 | 🔴 | 🔴 |
| **RESCEU^15^** | 🔴 | 🔴 | 🔴 | 🔴 | 🔴 | 🔴 | 🔴 | 🔴 | 🔴 | 🔴 | 🔴 |
| **Sharp 2022^23^** | 🔴 | 🔴 | 🔴 | 🔴 | 🔴 | 🔴 | 🔴 | 🔴 | 🔴 | 🔴 | 🔴 |
| **Tanner 2012^25^** | 🔴 | 🔴 | 🔴 | 🔴 | 🔴 | 🔴 | 🔴 | 🔴 | 🔴 | 🔴 | 🔴 |
| **Weir-Mccall 2022^26^** | 🔴 | 🔴 | 🔴 | 🔴 | 🔴 | 🔴 | 🔴 | 🔴 | 🔴 | 🔴 | 🔴 |
| **Youngs 2019^27^** | 🔴 | 🔴 | 🔴 | 🔴 | 🔴 | 🔴 | 🔴 | 🔴 | 🔴 | 🔴 | 🔴 |
| **Zhao 2014^28^** | 🔴 | 🔴 | 🔴 | 🔴 | 🔴 | 🔴 | 🔴 | 🔴 | 🔴 | 🔴 | 🔴 |
| **Studies assessing an elderly patient population** | | | | | | | | | | | |
| **Blackburn 2018^3^*** | 🔴 | 🔴 | 🔴 | 🔴 | 🔴 | 🔴 | 🔴 | 🔴 | 🔴 | 🔴 | 🔴 |
| **Chiavenna 2019^4^*** | 🔴 | 🔴 | 🔴 | 🔴 | 🔴 | 🔴 | 🔴 | 🔴 | 🔴 | 🔴 | 🔴 |
| **Fleming 2015^6^*** | 🔴 | 🔴 | 🔴 | 🔴 | 🔴 | 🔴 | 🔴 | 🔴 | 🔴 | 🔴 | 🔴 |
| **Hodgson 2020^11^*** | 🔴 | 🔴 | 🔴 | 🔴 | 🔴 | 🔴 | 🔴 | 🔴 | 🔴 | 🔴 | 🔴 |
| **Johannesen 2022^14^** | 🔴 | 🔴 | 🔴 | 🔴 | 🔴 | 🔴 | 🔴 | 🔴 | 🔴 | 🔴 | 🔴 |
| **Morbey 2018^17^*** | 🔴 | 🔴 | 🔴 | 🔴 | 🔴 | 🔴 | 🔴 | 🔴 | 🔴 | 🔴 | 🔴 |
| **Morbey 2017^16^*** | 🔴 | 🔴 | 🔴 | 🔴 | 🔴 | 🔴 | 🔴 | 🔴 | 🔴 | 🔴 | 🔴 |
| **Sharp 2022^23^** | 🔴 | 🔴 | 🔴 | 🔴 | 🔴 | 🔴 | 🔴 | 🔴 | 🔴 | 🔴 | 🔴 |
| **Tanner 2012^25^*** | 🔴 | 🔴 | 🔴 | 🔴 | 🔴 | 🔴 | 🔴 | 🔴 | 🔴 | 🔴 | 🔴 |
| **Zhao 2014^28^*** | 🔴 | 🔴 | 🔴 | 🔴 | 🔴 | 🔴 | 🔴 | 🔴 | 🔴 | 🔴 | 🔴 |
| **Studies assessing a co-morbid patient population** | | | | | | | | | | | |
| **Blackburn 2018^3^** | 🔴 | 🔴 | 🔴 | 🔴 | 🔴 | 🔴 | 🔴 | 🔴 | 🔴 | 🔴 | 🔴 |
| **Flight 2014^7^** | 🔴 | 🔴 | 🔴 | 🔴 | 🔴 | 🔴 | 🔴 | 🔴 | 🔴 | 🔴 | 🔴 |
| **Studies assessing an immunocompromised patient population** | | | | | | | | | | | |
| **Balassa 2019^1^** | 🔴 | 🔴 | 🔴 | 🔴 | 🔴 | 🔴 | 🔴 | 🔴 | 🔴 | 🔴 | 🔴 |
| **Gorcea 2017^9^** | 🔴 | 🔴 | 🔴 | 🔴 | 🔴 | 🔴 | 🔴 | 🔴 | 🔴 | 🔴 | 🔴 |
| **Inkster 2017^13^** | 🔴 | 🔴 | 🔴 | 🔴 | 🔴 | 🔴 | 🔴 | 🔴 | 🔴 | 🔴 | 🔴 |
| **Morris 2021^18^*** | 🔴 | 🔴 | 🔴 | 🔴 | 🔴 | 🔴 | 🔴 | 🔴 | 🔴 | 🔴 | 🔴 |
| **Partridge 2022^19^** | 🔴 | 🔴 | 🔴 | 🔴 | 🔴 | 🔴 | 🔴 | 🔴 | 🔴 | 🔴 | 🔴 |
| **Ponsford 2021^20^*** | 🔴 | 🔴 | 🔴 | 🔴 | 🔴 | 🔴 | 🔴 | 🔴 | 🔴 | 🔴 | 🔴 |
| Studies assessing a co-infected patient population | | | | | | | | | | | |
| **Chiavenna 2019^4^** | 🔴 | 🔴 | 🔴 | 🔴 | 🔴 | 🔴 | 🔴 | 🔴 | 🔴 | 🔴 | 🔴 |
| **Gadsby 2016^8^** | 🔴 | 🔴 | 🔴 | 🔴 | 🔴 | 🔴 | 🔴 | 🔴 | 🔴 | 🔴 | 🔴 |
| **Russell 2018^22^** | 🔴 | 🔴 | 🔴 | 🔴 | 🔴 | 🔴 | 🔴 | 🔴 | 🔴 | 🔴 | 🔴 |
| **Swets 2022^24^** | 🔴 | 🔴 | 🔴 | 🔴 | 🔴 | 🔴 | 🔴 | 🔴 | 🔴 | 🔴 | 🔴 |
| **Weir-Mccall 2022^26^** | 🔴 | 🔴 | 🔴 | 🔴 | 🔴 | 🔴 | 🔴 | 🔴 | 🔴 | 🔴 | 🔴 |

**Footnotes: ***studies reported on more than one patient population so have been considered twice in this table**. Key:** 🔴 low risk of bias,🔴 partial risk of bias,🔴 high risk of bias,🔴 not applicable

References

1. Balassa K, Salisbury R, Watson E, et al. Treatment stratification of respiratory syncytial virus infection in allogeneic stem cell transplantation. J Infect 2019;78:461-467.

2. Berry L, Lansbury L, Gale L, et al. Point of care testing of Influenza A/B and RSV in an adult respiratory assessment unit is associated with improvement in isolation practices and reduction in hospital length of stay. Journal of Medical Microbiology 2020;69:697-704.

3. Blackburn R, Zhao H, Pebody R, et al. Laboratory-confirmed respiratory infections as predictors of hospital admission for myocardial infarction and stroke: Time-series analysis of English data for 2004-2015. Clinical Infectious Diseases 2018;67(1):8-17.

4. Chiavenna C, Presanis AM, Charlett A, et al. Estimating age-stratified influenza-associated invasive pneumococcal disease in England: A time-series model based on population surveillance data. PLoS Medicine / Public Library of Science 2019;16:e1002829.

5. Dadhwal K, Stonham R, Breen H, et al. Severe COVID-19 pneumonia in an intensive care setting and comparisons with historic severe viral pneumonia due to other viruses. Clinical Respiratory Journal 2022;16(4):301-308.

6. Fleming DM, Taylor RJ, Lustig RL, et al. Modelling estimates of the burden of Respiratory Syncytial virus infection in adults and the elderly in the United Kingdom. BMC Infectious Diseases 2015;15(1) (no pagination).

7. Flight WG, Bright-Thomas RJ, Tilston P, et al. Incidence and clinical impact of respiratory viruses in adults with cystic fibrosis. Thorax 2014;69(3):247-253.

8. Gadsby NJ, Russell CD, McHugh MP, et al. Comprehensive molecular testing for respiratory pathogens in community-acquired pneumonia. Clinical Infectious Diseases 2016;62(7):817-823.

9. Gorcea CM, Tholouli E, Turner A, et al. Effective use of oral ribavirin for respiratory syncytial viral infections in allogeneic haematopoietic stem cell transplant recipients. J Hosp Infect 2017;95:214-217.

10. Green HK, Andrews N, Fleming D, et al. Mortality attributable to influenza in England and Wales prior to, during and after the 2009 pandemic. PLoS One 2013;8:e79360.

11. Hodgson D, Pebody R, Panovska-Griffiths J, et al. Evaluating the next generation of RSV intervention strategies: a mathematical modelling study and cost-effectiveness analysis. BMC Medicine 2020;18:348.

12. Hughes HE, Morbey R, Hughes TC, et al. Emergency department syndromic surveillance providing early warning of seasonal respiratory activity in England. Epidemiology and Infection 2016;144(5):1052-1064.

13. Inkster T, Ferguson K, Edwardson A, et al. Consecutive yearly outbreaks of respiratory syncytial virus in a haemato-oncology ward and efficacy of infection control measures. Journal of Hospital Infection 2017;96(4):353-359.

14. Johannesen CK, van Wijhe M, Tong S, et al. Age-Specific Estimates of Respiratory Syncytial Virus-Associated Hospitalizations in 6 European Countries: A Time Series Analysis. Journal of Infectious Diseases 2022;24:24.

15. Korsten K, Adriaenssens N, Coenen S, et al. Burden of respiratory syncytial virus infection in community-dwelling older adults in Europe (RESCEU): an international prospective cohort study. Eur Respir J 2021;57.

16. Morbey RA, Harcourt S, Pebody R, et al. The burden of seasonal respiratory infections on a national telehealth service in England. Epidemiology and Infection 2017;145(9):1922-1932.

17. Morbey RA, Elliot AJ, Harcourt S, et al. Estimating the burden on general practitioner services in England from increases in respiratory disease associated with seasonal respiratory pathogen activity. Epidemiology and Infection 2018;146(11):1389-1396.

18. Morris TC, Bird PW, Horvath-Papp E, et al. Xpert Xpress Flu/RSV: Validation and impact evaluation at a large UK hospital trust. J Med Virol 2021;93:5146-5151.

19. Partridge DG, Sori A, Green DJ, et al. Universal use of surgical masks is tolerated and prevents respiratory viral infection in stem cell transplant recipients. Journal of Hospital Infection 2022;119:182-186.

20. Ponsford MJ, Price C, Farewell D, et al. Increased Respiratory Viral Detection and Symptom Burden Among Patients with Primary Antibody Deficiency: Results from the BIPAD Study. Journal of Allergy and Clinical Immunology: In Practice 2021;9(2):735-744.e6.

21. Poole S, Brendish NJ, Clark TW. SARS-CoV-2 has displaced other seasonal respiratory viruses: Results from a prospective cohort study. Journal of Infection 2020;81:966-972.

22. Russell CD, Jones ME, O'Shea DT, et al. Challenges in the diagnosis of leptospirosis outwith endemic settings: A scottish single centre experience. Journal of the Royal College of Physicians of Edinburgh 2018;48(1):9-15.

23. Sharp A, Minaji M, Panagiotopoulos N, et al. Estimating the burden of adult hospital admissions due to RSV and other respiratory pathogens in England. Influenza Other Respir Viruses 2022;16:125-131.

24. Swets MC, Russell CD, Harrison EM, et al. SARS-CoV-2 co-infection with influenza viruses, respiratory syncytial virus, or adenoviruses. The Lancet 2022;399(10334):1463-1464.

25. Tanner H, Boxall E, Osman H. Respiratory viral infections during the 2009-2010 winter season in Central England, UK: Incidence and patterns of multiple virus co-infections. European Journal of Clinical Microbiology and Infectious Diseases 2012;31(11):3001-3006.

26. Weir-Mccall JR, Galea G, Mun Mak S, et al. Vascular Thrombosis in Severe COVID-19 Requiring Extracorporeal Membrane Oxygenation: A Multicenter Study. Critical Care Medicine 2022;50(4):624-632.

27. Youngs J, Iqbal Y, Glass S, et al. Implementation of the cobas Liat influenza point-of-care test into an emergency department during a high-incidence season: a retrospective evaluation following real-world implementation. Journal of Hospital Infection 2019;101:285-288.

28. Zhao H, Green H, Lackenby A, et al. A new laboratory-based surveillance system (Respiratory DataMart System) for influenza and other respiratory viruses in England: results and experience from 2009 to 2012. Euro Surveillance: Bulletin Europeen sur les Maladies Transmissibles = European Communicable Disease Bulletin 2014;19:23.

29. Agoti CN, Otieno JR, Ngama M, et al. Successive Respiratory Syncytial Virus Epidemics in Local Populations Arise from Multiple Variant Introductions, Providing Insights into Virus Persistence. J Virol 2015;89:11630-42.

30. Arnold D, Suri T, Hamilton F, et al. The role of viruses in the development of pleural infection. European Respiratory Journal 2019;54:PA3834.

31. Atabani SF, Wilson S, Overton-Lewis C, et al. Active screening and surveillance in the United Kingdom for Middle East respiratory syndrome coronavirus in returning travellers and pilgrims from the Middle East: a prospective descriptive study for the period 2013-2015. Int J Infect Dis 2016;47:10-4.

32. Bailey ES, Zemke JN, Choi JY, et al. A Mini-Review of Adverse Lung Transplant Outcomes Associated With Respiratory Viruses. Front Immunol 2019;10:2861.

33. Bhaskaran K, Hajat S, Armstrong B, et al. The effects of hourly differences in air pollution on the risk of myocardial infarction: case crossover analysis of the MINAP database. Bmj 2011;343:d5531.

34. Bouzid D, Vila J, Hansen G, et al. Systematic review on the association between respiratory virus real-time PCR cycle threshold values and clinical presentation or outcomes. J Antimicrob Chemother 2021;76:iii33-iii49.

35. Brittain-Long R, Andersson LM, Olofsson S, et al. Seasonal variations of 15 respiratory agents illustrated by the application of a multiplex polymerase chain reaction assay. Scand J Infect Dis 2012;44:9-17.

36. Broberg EK, Waris M, Johansen K, et al. Seasonality and geographical spread of respiratory syncytial virus epidemics in 15 European countries, 2010 to 2016. Euro Surveill 2018;23.

37. Broor S, Campbell H, Hirve S, et al. Leveraging the Global Influenza Surveillance and Response System for global respiratory syncytial virus surveillance-opportunities and challenges. Influenza Other Respir Viruses 2020;14:622-629.

38. Brunwasser SM, Snyder BM, Driscoll AJ, et al. Assessing the strength of evidence for a causal effect of respiratory syncytial virus lower respiratory tract infections on subsequent wheezing illness: a systematic review and meta-analysis. Lancet Respir Med 2020;8:795-806.

39. Bruyndonckx R, Coenen S, Butler C, et al. Respiratory syncytial virus and influenza virus infection in adult primary care patients: Association of age with prevalence, diagnostic features and illness course. Int J Infect Dis 2020;95:384-390.

40. Chadha M, Hirve S, Bancej C, et al. Human respiratory syncytial virus and influenza seasonality patterns-Early findings from the WHO global respiratory syncytial virus surveillance. Influenza Other Respir Viruses 2020;14:638-646.

41. Coughtrie AL, Morris DE, Anderson R, et al. Ecology and diversity in upper respiratory tract microbial population structures from a cross-sectional community swabbing study. J Med Microbiol 2018;67:1096-1108.

42. D'Anna SE, Maniscalco M, Carriero V, et al. Evaluation of Innate Immune Mediators Related to Respiratory Viruses in the Lung of Stable COPD Patients. J Clin Med 2020;9.

43. Dolby HW, Potey P, Wilder-Smith AB, et al. Histological Evidence of Pulmonary Microthrombosis and Vasculitis in Life-Threatening Respiratory Virus Diseases. Open Forum Infect Dis 2021;8:ofaa640.

44. Eyre TA, Peters L, Andersson MI, et al. Reduction in incidence of non-COVID-19 respiratory virus infection amongst haematology inpatients following UK social distancing measures. Br J Haematol 2021;195:194-197.

45. Falsey AR, McElhaney JE, Beran J, et al. Respiratory syncytial virus and other respiratory viral infections in older adults with moderate to severe influenza-like illness. J Infect Dis 2014;209:1873-81.

46. Falsey AR, Walsh EE, Esser MT, et al. Respiratory syncytial virus-associated illness in adults with advanced chronic obstructive pulmonary disease and/or congestive heart failure. J Med Virol 2019;91:65-71.

47. Falsey AR, Walsh EE, Osborne RH, et al. Comparative assessment of reported symptoms of influenza, respiratory syncytial virus, and human metapneumovirus infection during hospitalization and post-discharge assessed by Respiratory Intensity and Impact Questionnaire. Influenza Other Respir Viruses 2022;16:79-89.

48. Felt SA, Sun Y, Jozwik A, et al. Detection of respiratory syncytial virus defective genomes in nasal secretions is associated with distinct clinical outcomes. Nat Microbiol 2021;6:672-681.

49. Feng L, Li J, Yu HB, et al. Effects of cinnamaldehyde on anti-respiratory syncytial virus: A protocol of systematic review and meta-analysis. Medicine (Baltimore) 2020;99:e20266.

50. French CE, McKenzie BC, Coope C, et al. Risk of nosocomial respiratory syncytial virus infection and effectiveness of control measures to prevent transmission events: a systematic review. Influenza Other Respir Viruses 2016;10:268-90.

51. Gadsby NJ, McHugh MP, Forbes C, et al. Comparison of Unyvero P55 Pneumonia Cartridge, in-house PCR and culture for the identification of respiratory pathogens and antibiotic resistance in bronchoalveolar lavage fluids in the critical care setting. Eur J Clin Microbiol Infect Dis 2019;38:1171-1178.

52. Gaunt ER, Harvala H, McIntyre C, et al. Disease burden of the most commonly detected respiratory viruses in hospitalized patients calculated using the disability adjusted life year (DALY) model. J Clin Virol 2011;52:215-21.

53. Goka E, Vallely P, Mutton K, et al. Influenza A viruses dual and multiple infections with other respiratory viruses and risk of hospitalisation and mortality. Influenza Other Respir Viruses 2013;7:1079-87.

54. Goka EA, Vallely PJ, Mutton KJ, et al. Single, dual and multiple respiratory virus infections and risk of hospitalization and mortality. Epidemiol Infect 2015;143:37-47.

55. Goldstein EJ, Dhillon R, McCullough C, et al. Impact of implementing respiratory point-of-care testing in a regional haemato-oncology unit. J Hosp Infect 2020;106:20-24.

56. Goldstein EJ, Gunson RN. In-house validation of the cobas Liat influenza A/B and RSV assay for use with gargles, sputa and endotracheal secretions. J Hosp Infect 2019;101:289-291.

57. Guvenel A, Jozwik A, Ascough S, et al. Epitope-specific airway-resident CD4+ T cell dynamics during experimental human RSV infection. J Clin Invest 2020;130:523-538.

58. Haigh J, Cutino-Moguel MT, Wilks M, et al. A service evaluation of simultaneous near-patient testing for influenza, respiratory syncytial virus, Clostridium difficile and norovirus in a UK district general hospital. J Hosp Infect 2019;103:441-446.

59. Harcourt SE, Morbey RA, Smith GE, et al. Developing influenza and respiratory syncytial virus activity thresholds for syndromic surveillance in England. Epidemiol Infect 2019;147:e163.

60. Hardelid P, Pebody R, Andrews N. Mortality caused by influenza and respiratory syncytial virus by age group in England and Wales 1999-2010. Influenza Other Respir Viruses 2013;7:35-45.

61. Hodgson D, Atkins KE, Baguelin M, et al. Estimates for quality of life loss due to Respiratory Syncytial Virus. Influenza Other Respir Viruses 2020;14:19-27.

62. Hynicka LM, Ensor CR. Prophylaxis and treatment of respiratory syncytial virus in adult immunocompromised patients. Ann Pharmacother 2012;46:558-66.

63. Ieven M, Coenen S, Loens K, et al. Aetiology of lower respiratory tract infection in adults in primary care: a prospective study in 11 European countries. Clin Microbiol Infect 2018;24:1158-1163.

64. Jackson S, Peret TCT, Ziegler TT, et al. Results from the WHO external quality assessment for the respiratory syncytial virus pilot, 2016-17. Influenza Other Respir Viruses 2020;14:671-677.

65. Jansen A, van Deuren M, Miller J, et al. Prognosis of Good syndrome: mortality and morbidity of thymoma associated immunodeficiency in perspective. Clin Immunol 2016;171:12-17.

66. Johnson EK, Sylte D, Chaves SS, et al. Hospital utilization rates for influenza and RSV: a novel approach and critical assessment. Popul Health Metr 2021;19:31.

67. Kim YJ, Guthrie KA, Waghmare A, et al. Respiratory syncytial virus in hematopoietic cell transplant recipients: factors determining progression to lower respiratory tract disease. J Infect Dis 2014;209:1195-204.

68. Kirolos A, Christides A, Xian S, et al. A landscape review of the published research output relating to respiratory syncytial virus (RSV) in North & Central America and Europe between 2011-2015. J Glob Health 2019;9:010425.

69. Kombe IK, Munywoki PK, Baguelin M, et al. Model-based estimates of transmission of respiratory syncytial virus within households. Epidemics 2019;27:1-11.

70. Lam TT, Tang JW, Lai FY, et al. Comparative global epidemiology of influenza, respiratory syncytial and parainfluenza viruses, 2010-2015. J Infect 2019;79:373-382.

71. Lansbury L, Lim B, Baskaran V, et al. Co-infections in people with COVID-19: a systematic review and meta-analysis. J Infect 2020;81:266-275.

72. Lee N, Chan MC, Lui GC, et al. High Viral Load and Respiratory Failure in Adults Hospitalized for Respiratory Syncytial Virus Infections. J Infect Dis 2015;212:1237-40.

73. Li Y, Peterson ME, Campbell H, et al. Association of seasonal viral acute respiratory infection with pneumococcal disease: a systematic review of population-based studies. BMJ Open 2018;8:e019743.

74. Li Y, Reeves RM, Wang X, et al. Global patterns in monthly activity of influenza virus, respiratory syncytial virus, parainfluenza virus, and metapneumovirus: a systematic analysis. Lancet Glob Health 2019;7:e1031-e1045.

75. Li Y, Wang X, Msosa T, et al. The impact of the 2009 influenza pandemic on the seasonality of human respiratory syncytial virus: A systematic analysis. Influenza Other Respir Viruses 2021;15:804-812.

76. Ma X, Conrad T, Alchikh M, et al. Can we distinguish respiratory viral infections based on clinical features? A prospective pediatric cohort compared to systematic literature review. Rev Med Virol 2018;28:e1997.

77. Mahony J, Chong S, Bulir D, et al. Development of a sensitive loop-mediated isothermal amplification assay that provides specimen-to-result diagnosis of respiratory syncytial virus infection in 30 minutes. J Clin Microbiol 2013;51:2696-701.

78. Mandelia Y, Procop GW, Richter SS, et al. Optimal Timing of Repeat Multiplex Molecular Testing for Respiratory Viruses. J Clin Microbiol 2020;58.

79. Manikam L, Reed K, Venekamp RP, et al. Limited Evidence on the Management of Respiratory Tract Infections in Down's Syndrome: A Systematic Review. Pediatr Infect Dis J 2016;35:1075-9.

80. Mann A, Kalinova M, Catchpole A, et al. Late Breaking Abstract - Experimental Respiratory Syncytial Virus infection in adults 60-75 years. European Respiratory Journal 2020;56:4928.

81. Matias G, Taylor RJ, Haguinet F, et al. Modelling estimates of age-specific influenza-related hospitalisation and mortality in the United Kingdom. BMC Public Health 2016;16:481.

82. McNally JD, Sampson M, Matheson LA, et al. Vitamin D receptor (VDR) polymorphisms and severe RSV bronchiolitis: a systematic review and meta-analysis. Pediatr Pulmonol 2014;49:790-9.

83. Miller WT, Jr., Mickus TJ, Barbosa E, Jr., et al. CT of viral lower respiratory tract infections in adults: comparison among viral organisms and between viral and bacterial infections. AJR Am J Roentgenol 2011;197:1088-95.

84. Millership S, Cummins A. Oseltamivir in influenza outbreaks in care homes: challenges and benefits of use in the real world. J Hosp Infect 2015;90:299-303.

85. Mohammed NI, Everard ML, Ayres JG, et al. A Preliminary Assessment of the Role of Ambient Nitric Oxide Exposure in Hospitalization with Respiratory Syncytial Virus Bronchiolitis. Int J Environ Res Public Health 2016;13.

86. Mollers M, Barnadas C, Broberg EK, et al. Current practices for respiratory syncytial virus surveillance across the EU/EEA Member States, 2017. Euro Surveill 2019;24.

87. Moore C, Jones R. The use of coroner's autopsy reports to validate the use of targeted swabbing rather than tissue collection for rapid confirmation of virological causes of sudden death in the community. J Clin Virol 2015;63:59-62.

88. Nicoli EJ, Trotter CL, Turner KM, et al. Influenza and RSV make a modest contribution to invasive pneumococcal disease incidence in the UK. J Infect 2013;66:512-20.

89. Nicholson KG, Abrams KR, Batham S, et al. Randomised controlled trial and health economic evaluation of the impact of diagnostic testing for influenza, respiratory syncytial virus and Streptococcus pneumoniae infection on the management of acute admissions in the elderly and high-risk 18- to 64-year-olds. Health Technol Assess 2014;18:1-274, vii-viii.

90. Obando-Pacheco P, Justicia-Grande AJ, Rivero-Calle I, et al. Respiratory syncytial virus seasonality: a global overview. J Infect Dis 2018;217:1356-1364.

91. Olchanski N, Hansen RN, Pope E, et al. Palivizumab Prophylaxis for Respiratory Syncytial Virus: Examining the Evidence Around Value. Open Forum Infect Dis 2018;5:ofy031.

92. Osborne RH, Nelson L, Elsworth GR, et al. PIN70 Psychometric Evaluation of the Respiratory Infection Intensity and Impact Questionnaire Version 2 (RiiQ&#xc2;&#x2122;v2) Symptom Scores for Respiratory Syncytial Virus. Value in Health 2021;24:S119.

93. Paba P, Farchi F, Mortati E, et al. Screening of respiratory pathogens by Respiratory Multi Well System (MWS) r-gene™ assay in hospitalized patients. New Microbiol 2014;37:231-6.

94. Pando R, Stern S, Nemet I, et al. Diversity in the Circulation of Influenza A(H3N2) Viruses in the Northern Hemisphere in the 2018-19 Season. Vaccines (Basel) 2021;9.

95. Papadopoulos NG, Christodoulou I, Rohde G, et al. Viruses and bacteria in acute asthma exacerbations--a GA² LEN-DARE systematic review. Allergy 2011;66:458-68.

96. Permpalung N, Mahoney MV, McCoy C, et al. Clinical characteristics and treatment outcomes among respiratory syncytial virus (RSV)-infected hematologic malignancy and hematopoietic stem cell transplant recipients receiving palivizumab. Leuk Lymphoma 2019;60:85-91.

97. Price RHM, Graham C, Ramalingam S. Association between viral seasonality and meteorological factors. Sci Rep 2019;9:929.

98. Richardson L, Brite J, Del Castillo M, et al. Comparison of respiratory virus shedding by conventional and molecular testing methods in patients with haematological malignancy. Clin Microbiol Infect 2016;22:380.e1-380.e7.

99. Rose JJ, Voora D, Cyr DD, et al. Gene Expression Profiles Link Respiratory Viral Infection, Platelet Response to Aspirin, and Acute Myocardial Infarction. PLoS One 2015;10:e0132259.

100. Russell CD, Unger SA, Walton M, et al. The Human Immune Response to Respiratory Syncytial Virus Infection. Clin Microbiol Rev 2017;30:481-502.

101. Shi T, Arnott A, Semogas I, et al. The Etiological Role of Common Respiratory Viruses in Acute Respiratory Infections in Older Adults: A Systematic Review and Meta-analysis. J Infect Dis 2020;222:S563-s569.

102. Shi T, Denouel A, Tietjen AK, et al. Global Disease Burden Estimates of Respiratory Syncytial Virus-Associated Acute Respiratory Infection in Older Adults in 2015: A Systematic Review and Meta-Analysis. J Infect Dis 2020;222:S577-s583.

103. Shi T, Vennard S, Jasiewicz F, et al. Disease Burden Estimates of Respiratory Syncytial Virus related Acute Respiratory Infections in Adults With Comorbidity: A Systematic Review and Meta-Analysis. J Infect Dis 2022;226:S17-s21.

104. Smit PM, Limper M, van Gorp EC, et al. Adult outpatient experience of the 2009 H1N1 pandemic: clinical course, pathogens, and evaluation of case definitions. J Infect 2011;62:371-8.

105. Smith S, Morbey R, Pebody RG, et al. Retrospective Observational Study of Atypical Winter Respiratory Illness Season Using Real-Time Syndromic Surveillance, England, 2014-15. Emerg Infect Dis 2017;23:1834-1842.

106. Steensels D, Reynders M, Descheemaeker P, et al. Performance evaluation of direct fluorescent antibody, Focus Diagnostics Simplexa™ Flu A/B & RSV and multi-parameter customized respiratory Taqman® array card in immunocompromised patients. J Virol Methods 2017;245:61-65.

107. Sundaram ME, Meece JK, Sifakis F, et al. Medically attended respiratory syncytial virus infections in adults aged ≥ 50 years: clinical characteristics and outcomes. Clin Infect Dis 2014;58:342-9.

108. Sundell N, Andersson LM, Brittain-Long R, et al. A four year seasonal survey of the relationship between outdoor climate and epidemiology of viral respiratory tract infections in a temperate climate. J Clin Virol 2016;84:59-63.

109. Szabo SM, Levy AR, Gooch KL, et al. Elevated risk of asthma after hospitalization for respiratory syncytial virus infection in infancy. Paediatr Respir Rev 2013;13 Suppl 2:S9-15.

110. Tang JW, Blount J, Bradley C, et al. Impact of a poorly performing point-of-care test during the 2017-2018 influenza season. J Infect 2019;78:249-259.

111. Tanner AR, Dorey RB, Brendish NJ, et al. Influenza vaccination: protecting the most vulnerable. Eur Respir Rev 2021;30.

112. Taplitz R, Maziarz RT, Mulroney C, et al. Incidence and Impact of Community Respiratory Viral Infection (CRV) in Haploidentical and Matched Sibling Donors Receiving Post-Transplant Cyclophosphamide (PTCy): A CIBMTR Analysis. Biology of Blood and Marrow Transplantation 2020;26:S71-S72.

113. Thorburn F, Bennett S, Modha S, et al. The use of next generation sequencing in the diagnosis and typing of respiratory infections. J Clin Virol 2015;69:96-100.

114. Thornton HV, Turner KME, Harrison S, et al. Assessing the potential of upper respiratory tract point-of-care testing: a systematic review of the prognostic significance of upper respiratory tract microbes. Clin Microbiol Infect 2019;25:1339-1346.

115. Todkill D, Loveridge P, Elliot AJ, et al. Utility of Ambulance Data for Real-Time Syndromic Surveillance: A Pilot in the West Midlands Region, United Kingdom. Prehosp Disaster Med 2017;32:667-672.

116. Treskova M, Pozo-Martin F, Scholz S, et al. Assessment of the Effects of Active Immunisation against Respiratory Syncytial Virus (RSV) using Decision-Analytic Models: A Systematic Review with a Focus on Vaccination Strategies, Modelling Methods and Input Data. Pharmacoeconomics 2021;39:287-315.

117. van Summeren J, Meijer A, Aspelund G, et al. Low levels of respiratory syncytial virus activity in Europe during the 2020/21 season: what can we expect in the coming summer and autumn/winter? Euro Surveill 2021;26.

118. Verbakel JY, Matheeussen V, Loens K, et al. Performance and ease of use of a molecular point-of-care test for influenza A/B and RSV in patients presenting to primary care. Eur J Clin Microbiol Infect Dis 2020;39:1453-1460.

119. Visconti MR, Usategui-Martín R, Ralston SH. Antibody Response to Paramyxoviruses in Paget's Disease of Bone. Calcif Tissue Int 2017;101:141-147.

120. Vos LM, Bruyndonckx R, Zuithoff NPA, et al. Lower respiratory tract infection in the community: associations between viral aetiology and illness course. Clin Microbiol Infect 2021;27:96-104.

121. Waghmare A, Campbell AP, Xie H, et al. Respiratory syncytial virus lower respiratory disease in hematopoietic cell transplant recipients: viral RNA detection in blood, antiviral treatment, and clinical outcomes. Clin Infect Dis 2013;57:1731-41.

122. Walsh EE, Peterson DR, Kalkanoglu AE, et al. Viral shedding and immune responses to respiratory syncytial virus infection in older adults. J Infect Dis 2013;207:1424-32.

123. Walsh EE, Shin JH, Falsey AR. Clinical impact of human coronaviruses 229E and OC43 infection in diverse adult populations. J Infect Dis 2013;208:1634-42.

124. Wang X, Li Y, Mei X, et al. Global hospital admissions and in-hospital mortality associated with all-cause and virus-specific acute lower respiratory infections in children and adolescents aged 5-19 years between 1995 and 2019: a systematic review and modelling study. BMJ Glob Health 2021;6.

125. Waterlow NR, Flasche S, Minter A, et al. Competition between RSV and influenza: Limits of modelling inference from surveillance data. Epidemics 2021;35:100460.

126. Wilcox CR, Calvert A, Metz J, et al. Attitudes of Pregnant Women and Healthcare Professionals Toward Clinical Trials and Routine Implementation of Antenatal Vaccination Against Respiratory Syncytial Virus: A Multicenter Questionnaire Study. Pediatr Infect Dis J 2019;38:944-951.

127. Wiseman DJ, Kamal F, Finney L, et al. Respiratory Syncytial Virus (RSV) Detection Is Associated with an Increased Inflammatory Response in Stable (non-Exacerbating) Chronic Obstructive Pulmonary Disease (COPD) Patients. A24. COPD: THERAPY, IMAGING, AND MOLECULAR MARKERS: American Thoracic Society, 2019:A1129-A1129.

128. Wiseman DJ, Thwaites RS, Drysdale SB, et al. Immunological and Inflammatory Biomarkers of Susceptibility and Severity in Adult Respiratory Syncytial Virus Infections. J Infect Dis 2020;222:S584-s591.

129. Wong SS, Yu JW, Wong KT, et al. Initial radiographic features as outcome predictor of adult respiratory syncytial virus respiratory tract infection. AJR Am J Roentgenol 2014;203:280-6.

130. Xu Y, Lewandowski K, Downs LO, et al. Nanopore metagenomic sequencing of influenza virus directly from respiratory samples: diagnosis, drug resistance and nosocomial transmission, United Kingdom, 2018/19 influenza season. Euro Surveill 2021;26.

131. Zeevat F, Luttjeboer J, Beutels P, et al. PIN25 EXPLORATORY ANALYSIS OF THE ECONOMICALLY JUSTIFIABLE PRICE OF AN RSV VACCINE FOR THE ELDERLY IN THE NETHERLANDS AND THE UNITED KINGDOM. Value in Health 2019;22:S643-S644.

132. Zeevat F, Luttjeboer J, Paulissen JHJ, et al. Exploratory Analysis of the Economically Justifiable Price of a Hypothetical RSV Vaccine for Older Adults in the Netherlands and the United Kingdom. J Infect Dis 2022;226:S102-s109.

133. Zwaans WA, Mallia P, van Winden ME, et al. The relevance of respiratory viral infections in the exacerbations of chronic obstructive pulmonary disease—a systematic review. J Clin Virol 2014;61:181-8.

134. Baird D, Muir A, Logan L, et al. Validation of the NeuMoDx™ SARS-CoV-2 assay with COPAN eNAT® and E&O Viral PCR Sample Solution collection media types in comparison with other validated SARS-CoV-2 RNA assays. Int J Infect Dis 2022;122:864-866.

135. Bernstein DI, Mejias A, Rath B, et al. Diagnostic Accuracy of Commercially Available Tests for Respiratory Syncytial Virus: A Scoping Literature Review in the COVID-19 Era. medRxiv 2022:2022.02.14.22270927.

136. Bernstein DI, Mejias A, Rath B, et al. Summarizing Study Characteristics and Diagnostic Performance of Commercially Available Tests for Respiratory Syncytial Virus: A Scoping Literature Review in the COVID-19 Era. J Appl Lab Med 2022.

137. Campbell TM, Liu Z, Zhang Q, et al. Respiratory viral infections in otherwise healthy humans with inherited IRF7 deficiency. J Exp Med 2022;219.

138. Chapman T, Etel E, Moudgil H, et al. Another aspect of COVID pandemic: where has all the Flu gone? European Respiratory Journal 2021;58:PA3255.

139. Chellapuri A, Smitheman M, Chappell JG, et al. Human parainfluenza 2 &amp; 4: Clinical and genetic epidemiology in the UK, 2013-2017, reveals distinct disease features and co-circulating genomic subtypes. Influenza Other Respir Viruses 2022;16:1122-1132.

140. Chellapuri A, Smitheman M, Chappell JG, et al. Human Parainfluenza 2 & 4: clinical and genetic epidemiology in the UK, 2013-2017, reveals distinct disease features and co-circulating genomic subtypes. medRxiv 2022:2022.01.20.22269219.

141. Falsey AR, Walsh EE, House S, et al. Risk Factors and Medical Resource Utilization of Respiratory Syncytial Virus, Human Metapneumovirus, and Influenza-Related Hospitalizations in Adults-A Global Study During the 2017-2019 Epidemic Seasons (Hospitalized Acute Respiratory Tract Infection [HARTI] Study). Open Forum Infect Dis 2021;8:ofab491.

142. Flynn MF, Kelly M, Dooley JSG. Nasopharyngeal Swabs vs. Nasal Aspirates for Respiratory Virus Detection: A Systematic Review. Pathogens 2021;10.

143. Jiang XW, Huang TS, Xie L, et al. Development of a diagnostic assay by three-tube multiplex real-time PCR for simultaneous detection of nine microorganisms causing acute respiratory infections. Sci Rep 2022;12:13306.

144. Korsten K, Adriaenssens N, Coenen S, et al. World Health Organization Influenza-Like Illness Underestimates the Burden of Respiratory Syncytial Virus Infection in Community-Dwelling Older Adults. J Infect Dis 2022;226:S71-s78.

145. Krauer F, Fjelde TE, Koltai M, et al. Estimating RSV seasonality from pandemic disruptions: a modelling study. medRxiv 2022:2022.06.18.22276591.

146. Li Y, Wang X, Broberg EK, et al. Seasonality of respiratory syncytial virus and its association with meteorological factors in 13 European countries, week 40 2010 to week 39 2019. Euro Surveill 2022;27.

147. Li Y, Wang X, Cong B, et al. Understanding the Potential Drivers for Respiratory Syncytial Virus Rebound During the Coronavirus Disease 2019 Pandemic. J Infect Dis 2022;225:957-964.

148. Lin GL, Drysdale SB, Snape MD, et al. Publisher Correction: Distinct patterns of within-host virus populations between two subgroups of human respiratory syncytial virus. Nat Commun 2021;12:5971.

149. Magron A. Usage optimal des immunoglobulines en transplantation d'organes solides - Rapport en soutien au guide d'usage optimal, 2022.

150. Matheeussen V, Loens K, Kuijstermans M, et al. Diagnostic performance of the Idylla™ respiratory panel for molecular detection of influenza A/B in patients presenting to primary care with influenza-like illness during 3 consecutive influenza seasons. J Clin Virol 2021;144:104998.

151. Mulenga H, Musvosvi M, Mendelsohn SC, et al. Longitudinal Dynamics of a Blood Transcriptomic Signature of Tuberculosis. Am J Respir Crit Care Med 2021;204:1463-1472.

152. Popowitch EB, Kaplan S, Wu Z, et al. Comparative Performance of the Luminex NxTAG Respiratory Pathogen Panel, GenMark eSensor Respiratory Viral Panel, and BioFire FilmArray Respiratory Panel. Microbiol Spectr 2022;10:e0124822.

153. Shaterian N, Abdi F, Atarodi Kashani Z, et al. Facemask and Respirator in Reducing the Spread of Respiratory Viruses; a Systematic Review. Arch Acad Emerg Med 2021;9:e56.

154. Stewart A, Sinclair E, Ng JC, et al. Pandemic, Epidemic, Endemic: B Cell Repertoire Analysis Reveals Unique Anti-Viral Responses to SARS-CoV-2, Ebola and Respiratory Syncytial Virus. Front Immunol 2022;13:807104.

155. Green HK, Ellis J, Galiano M, et al. Critical care surveillance: insights into the impact of the 2010/11 influenza season relative to the 2009/10 pandemic season in England. Euro Surveill 2013;18.

156. Kmet LM, Cook LS, Lee RC. Standard quality assessment criteria for evaluating primary research papers from a variety of fields. 2004.
